# MycoNews 2023: Editorial, news, reports, awards, personalia, and book news

**DOI:** 10.1186/s43008-024-00139-8

**Published:** 2024-02-05

**Authors:** David L. Hawksworth

**Affiliations:** 1 Trait Diversity and Function, Royal Botanic Gardens, Kew, Surrey TW9 3DS UK; 2 Department of Life Sciences, The Natural History Museum, Cromwell Road, London, SW7 5BD UK; 3 Jilin Agricultural University, Changchun, 130118 Jilin Province China; 4 Geography and Environmental Science, University of Southampton, Highfield Campus, Southampton, SO17 1BJ UK

**Keywords:** Birthday greetings, Book reviews, International mycological congresses, IMC12, Meeting reports, Nomenclatural stability, Obituaries

## Abstract

This fifth annual edition of *MycoNews* starts with an editorial on the critical importance of International Mycological Congresses (IMCs) to the health of mycology. Items on Counting down to IMC12, the State of the World’s Plants and Fungi 2023, and progress towards Improving nomenclatural stability in medically important fungi follow. Reports are provided of several mycological meetings in 2023: the Asian Mycological Congress, XIX Congress of European Mycologists, a meeting of European Mycological Groups and Societies, the XI Latin American Mycological Congress, Westerdijk Spring Symposium on Fungal Evolution, the Brazilian Society of Mycology, the Annual Meeting of the Mycological Society of China, and the Fifth Iranian Mycological Congress. Information is provided on how to make nominations for the various IMA Awards due to be presented at IMC12 in August, the new Future is Fungi Award launched in 2023, and the Adel-Azeem and Stamets Award for work on *Psilocybe* in Africa. The Westerdijk Fungal Biodiversity Institute Awards for 2023 were made to Andrey Yurkov and Cathie Aime and the citations to those awards are provided. We include tributes to the passing of two eminent mycologists, Lorelei Norvell and Takashi Matsushima, and also send birthday greetings to Bryce Kendrick who turned 90, and Maria Ławrynowicz, Yu Li, and Anthony Whalley who all became octogenarians. Reviews of seven mycological books published in 2022–2023 are included in the Book News section.

## THE CRITICAL IMPORTANCE OF IMC’S TO THE HEALTH OF MYCOLOGY

With the prospect of the delayed 12th International Mycological Congress (IMC12) at last becoming a reality on 11–15 August 2024 in Maastricht, The Netherlands, it is important for mycologists to appreciate why these Congresses are so important for the health of the discipline, especially in the current electronic age.

The first Congress (IMC1) was held at the University of Exeter, UK in September 1971. It was inspired by Geoffrey C. Ainsworth (1905–1998), who had retired as Director of what was then the Commonwealth Mycological Institute at Kew in 1968. It was there that the International Mycological Association (IMA) was launched by representatives of 21 of world’s largest mycological societies; I recall standing in the audience when the decision was announced, and feeling something of a tingle in my spine. It was a truly momentous event. The congress attracted 905 participants from 45 countries, which in itself testified to its’ need. I had been on the Institute’s staff for barely two years, but ended up running a pre-congress field excursion for lichenologists and organizing a symposium on chemotaxonomy.

**Fig. 1:**
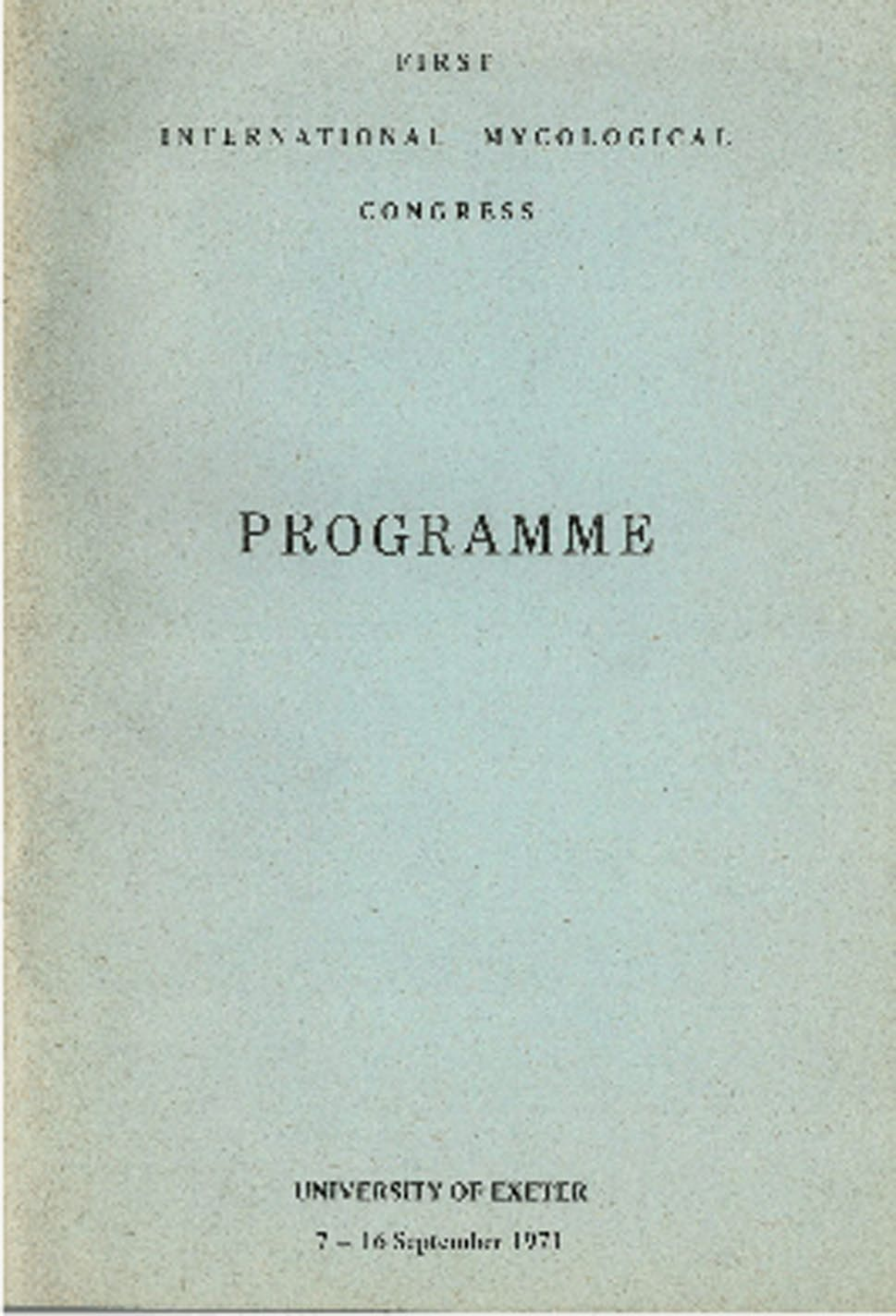
IMC1 (Exeter 1971) Programme Booklet

A major motivation for the Congress was a need to have one international event where mycologists of diverse interests in fungi were able to get together to learn of the latest research and exchange views with colleagues (Fig. 1). It was an enormously stimulating, even mind-blowing in modern jargon. It was all rather overwhelming for a youth who had gained a PhD only in 1970 for a taxonomic study on a single genus and who had never been to an event with more than 100 mycologists in a single room. It was amazing to see in reality so many of the “names” I had come to be familiar with while checking the metre-long sheaves of galley proofs for the sixth edition of *Ainsworth & Bisby’s Dictionary of the Fungi* (which was published for the Congress).

But more important was the chance not just to hear the leading mycologists of the day speak, something enormously stimulating in itself, but the chance to timidly question and seek opinions of some of those “names”, especially during informal social events and during field events.

Historically, the rules that governed the naming of fungi had been controlled by decisions made at International Botanical Congresses (IBCs). There was dissatisfaction over several issues, and there were two special meetings on Mycological Nomenclature organized. A series of committees was then established under a Nomenclatural Secretariat and charged to bring recommendations to IMC2 in Tampa, Florida in 1977 for further discussion. At last mycologists were starting to take responsibility for their own nomenclatural destiny, and a series of proposals was made to the next International Botanical Congress in 1981; amongst those adopted were the abolition of so-called “later starting points” and new provisions for the naming of morphs of pleomorphic fungi.

Such nomenclatural discussions have become an increasing feature of IMCs, and in 2017 the International Botanical Congress in Shenzhen, China, voted to permit decisions on rules pertaining only to fungal organisms to be decided on at IMCs not IBCs. This was a major step forward meaning that at last mycologists are alone responsible for their own destinies in issues of nomenclature. Any changes in the rules relating to fungi can now be influenced by and reflect those of mycologists voting on them at IMCs, and not leaving matters to the few willing and able to afford to attend the week-log nomenclatural meetings held before each IBC.

**Fig. 2:**
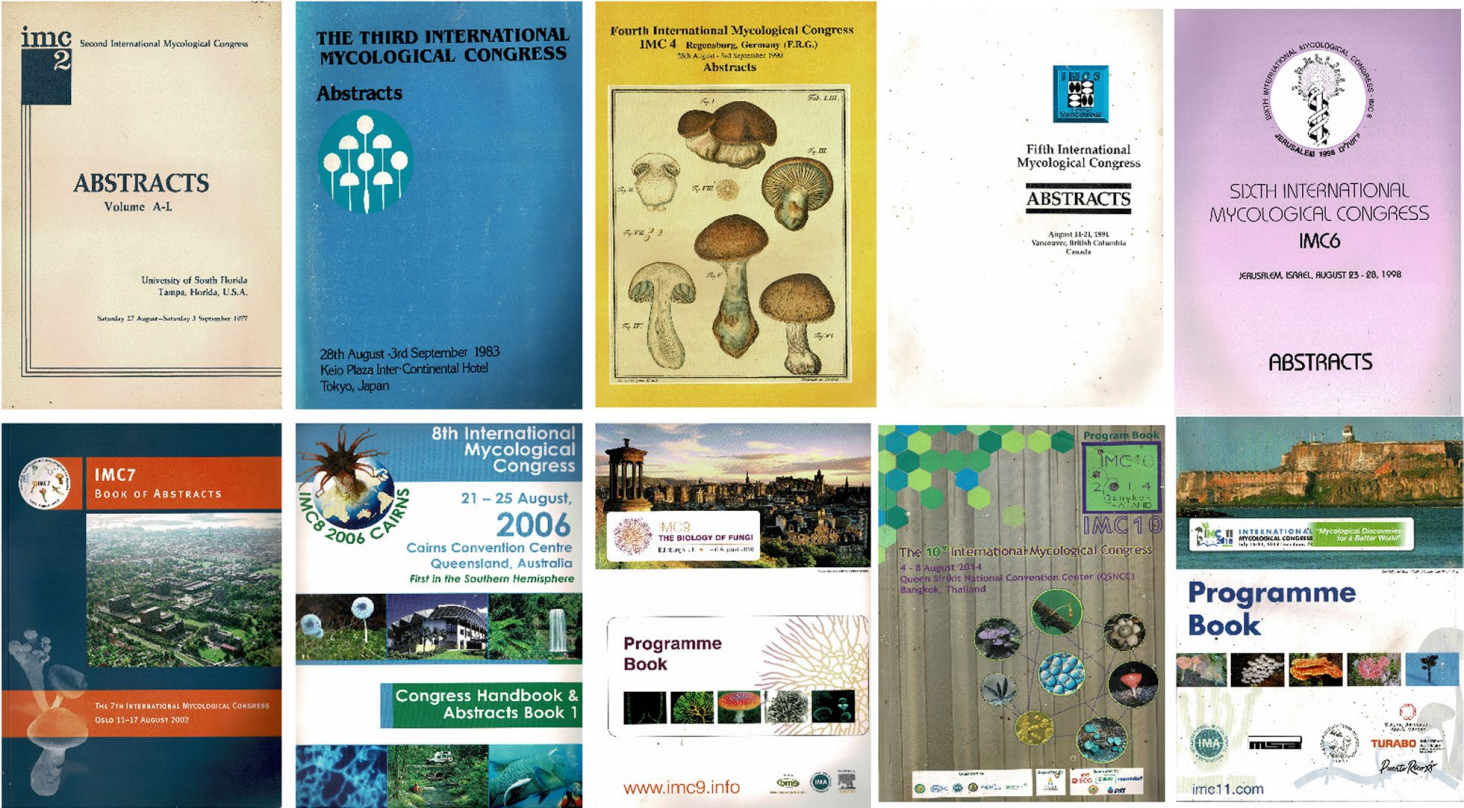
Abstract books for IMC 2 (Tampa 1977), IMC3 (Tokyo 1983), IMC4 (Regensburg 1990), IMC5 (Vancouver 1994), IMC6 (Jerusalem1998), IMC7 (Oslo 2002), IMC8 (Cairns, 2006), IMC9 (Edinburgh, 2010), IMC10 (Bangkok, 2014), and IMC11 (Puerto Rico, 2018)

Each IMC (Fig. 2) had its own special flavours and highlights, and detailed information on all up to and including IMC8 (Cairns 2006) and with many photographs of individuals involved, are provided by the late Emory G. Simmons (1920–2013), chairman of the Executive Committee organizing IMC2 and IMA Vice-President for 1983–1990 (Simmons 2010).

What all IMCs have retained, at least for me personally, is a feeling of excitement at such a key event, with the opportunity to meet, learn, and question leading workers of the day, discover what is happening across the whole spectrum of pure and applied aspects of the study of fungi worldwide, and not least to renew acquaintances and enjoy splendid informal social events. It is also an opportunity for younger mycologists particularly to showcase their own work, even if rather apprehensively as a poster or brief offered paper, but then benefit from comments and advice from those that see or hear it.

In 2024, we see a subject that is becoming increasingly diverse, with an unparalleled number of publications appearing electronically in seemingly evermore dedicated journals. It is now hardly possible to keep abreast of work except in the narrowest of fields, even if spending several hours a day looking at computer screens rather than working productively on original contributions. At the same time, similar technologies are being used in different parts of our discipline with advances pertinent to our work which we may not be aware of but could benefit from. There are more and yet more meetings organized virtually which can be most valuable, but they tend to be narrowly focussed and a video link hardly compares with the chance to quiz someone over a glass or two of beer—where they may divulge points they might not yet wish to broadcast.

So, if you have not already made your plans to attend IMC12 this coming August, I would like to urge you to do so, whether you are a ‘professional’ or not. This promises to be an especially momentous occasion, as you will see from the following pages. We are entering an exciting time for mycology in which the world is re-kindling an interest in fungi as never before. This IMC is an opportunity for each of us to make sure we are at the forefront of what is taking place in our science, to be part of that worldwide movement, and to consider how we might capitalize on that wider interest.

- **David L Hawksworth**
- *Hon. President, IMA*
- (d.hawksworth@kew.org)

## COUNTING DOWN TO IMC12

**Fig. 3:**
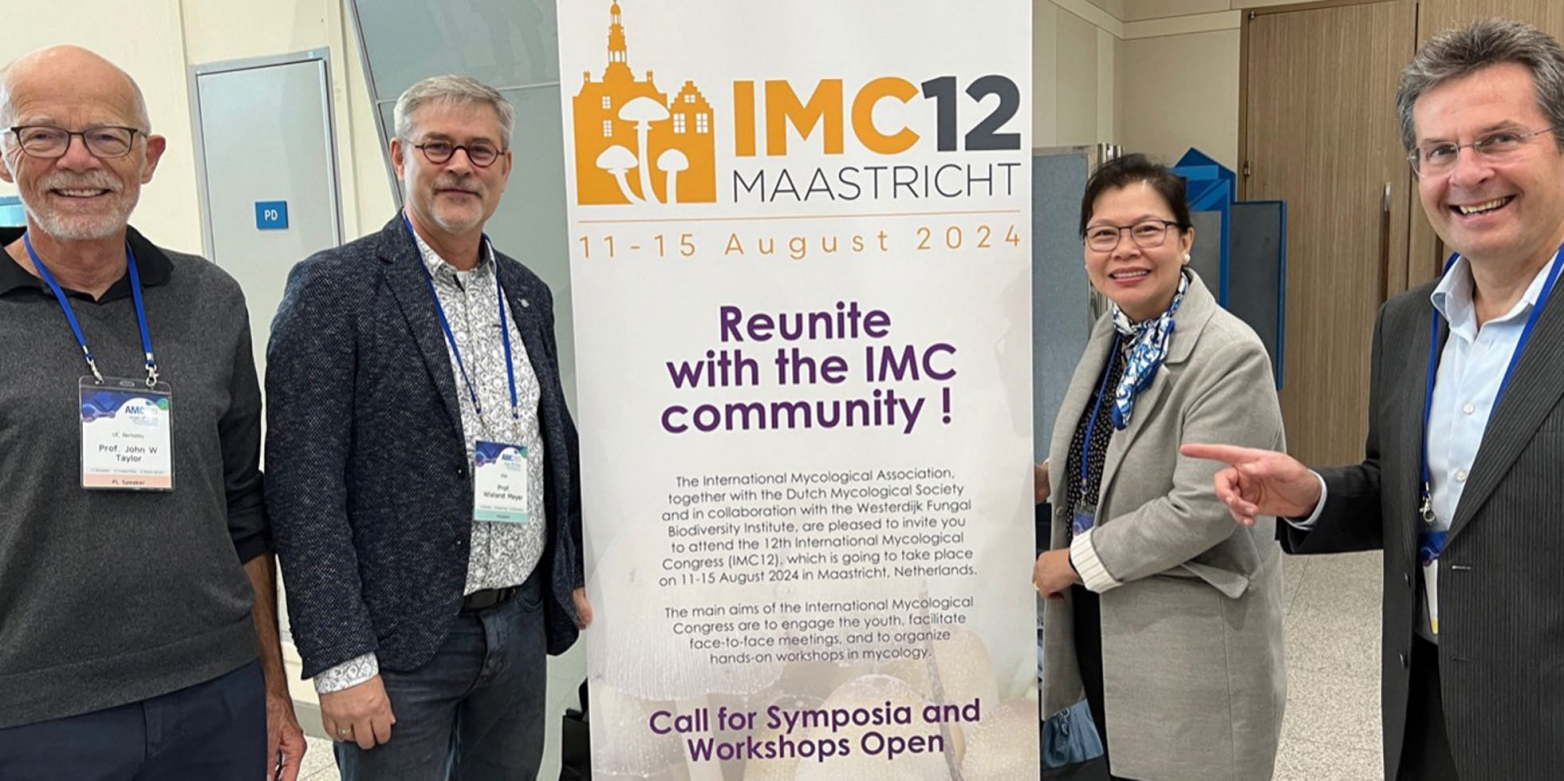
IMC12 Banner with (left to right) John W. Taylor, Weiland Meyer, Jennifer Luangsa-ard, and Pedro W Crous at the Asian Mycological Congress

The International Mycological Congress (IMC12; Fig. 3) in Maastricht is now just a few months away (11–15 August 2024)!

The meeting will bring together mycologists from all around the globe to discuss their latest research and current developments in fungal research.

We are being hosted by the Netherlands Mycological Society and the Westerdijk Fungal Biodiversity Institute, who have set up an international scientific committee to develop a programme to incorporate all fields of mycology around the theme “Fungal Biology and Applications”. The IMC12 scientific programme, satellite meetings, and workshops will be rich with talks, posters and opportunities for discussions with links to all aspects of mycology.

Maastricht is one of Europe’s most impressive cities with a rich history. This four-day meeting will include keynote lectures by scientific leaders, bridging sessions and workshops in seven themes:

- Cell biology, biochemistry and physiology.
- Environment, ecology and interactions.
- Evolution, biodiversity and systematics.
- Fungal pathogenesis and disease control.
- Genomics, genetics and molecular biology.
- Applied mycology.
- Nomenclature.

The keynote speakers are all now in place, and chairs have been appointed for each of the three symposia to be held within each theme (see *IMA Fungus* **14** (1): 3–6, 2023).

***The call is open for the submission of symposium and workshop proposals, but not for much longer!*** Go to https://imc12.org/symposium-proposal-submission-and-guidelines/ for the latest information.

For the latest news on IMC12, see imc_reply@kenes-group.com.

We look forward to welcoming you in Maastricht in person!

- **Pedro W. Crous**
- *Chair, IMC12*
- (p.crous@wi.knaw.nl)

## STATE OF THE WORLD’S PLANTS AND FUNGI

**Fig. 4:**
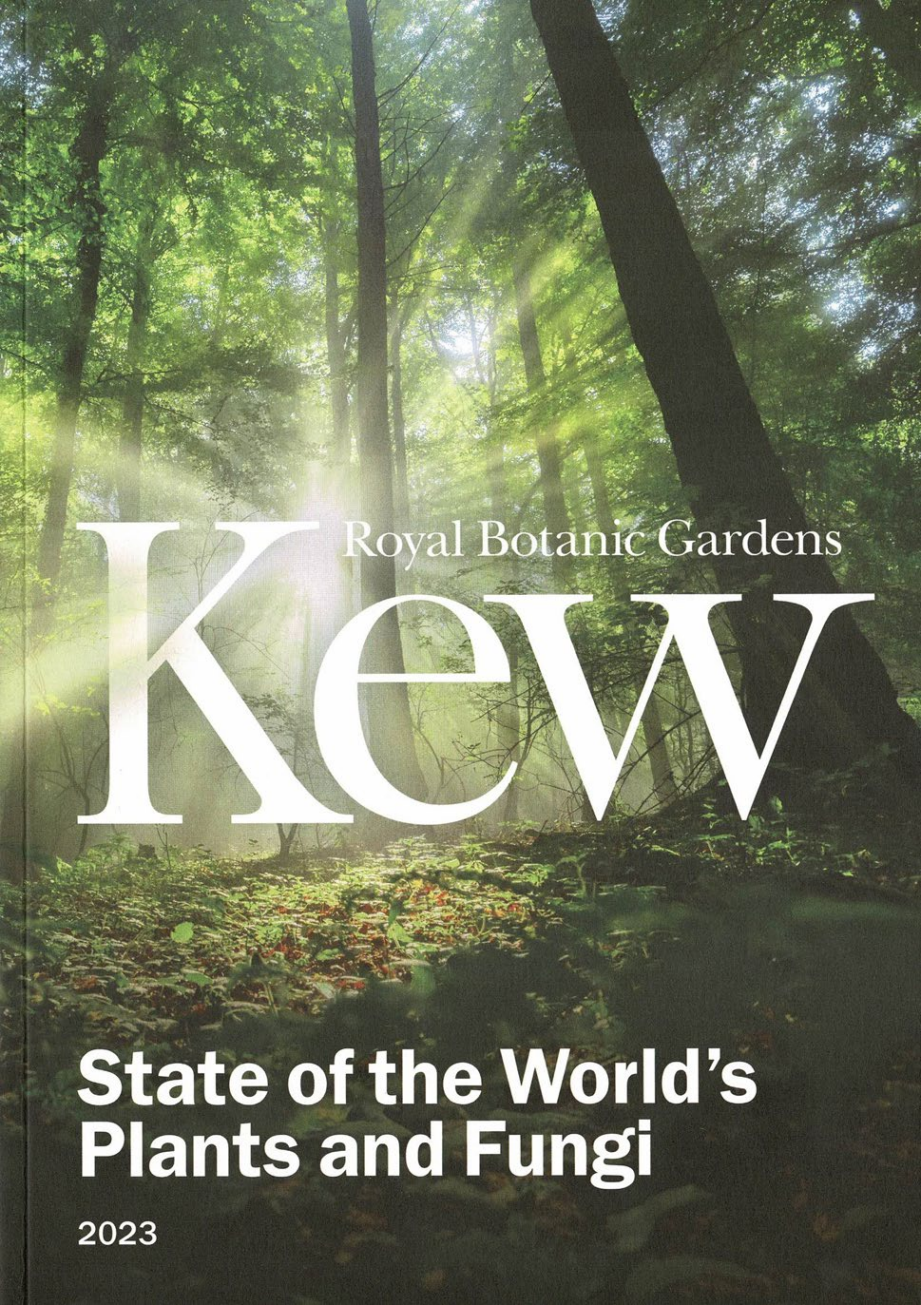
State of the World’s Plants and Fungi (2023)

A three-day meeting was held at the Royal Botanic Gardens Kew on 11–13 October 2023 to launch the 90 page profusely illustrated 2023 assessment of the *State of World’s Plants and Fungi,* subtitled *Tackling the Nature Emergency: evidence, gaps, and priorities* (Antonelli et al. 2023; Fig. 4); it can be downloaded free of charge as a pdf from https://www.kew.org/science/state-of-the-worlds-plants-and-fungi. This, the fifth assessment in the series, relies on two major recent advances, release of the world checklist of vascular plants which includes distributional information for all 350,386 accepted described species, and the wealth of new information on fungal diversity arising from environmental DNA, especially from soil samples. The report involved 200 scientists from 102 institutions in 30 countries spread around the world.

Unlike its predecessors, the report relies on a collection of articles on global plant diversity published in the *New Phytologist*, and a separate multi-authored review of global fungal diversity which appeared in the *Annual Review of Environment and Resources* (Niskanen et al. 2023).

Chapters of especial interest to mycologists in the report are ones on Finding out the fundamentals of fungi, Making sense of patterns of diversity, and Conserving forgotten fungi. The assessment on global species numbers of fungi comes out with an estimate of 2.5 million, compared with 450,000 plants and 8.5 million invertebrates (Niskanen et al. 2023). Thus it seems the various fungal estimates are now settling around a similar figure.

Amongst speakers at the symposium were mycologists Lynne Boddy, Catherine Aime, Anders Dahlberg, Giuliana Furci, and Aidas M Vasco Palacios. Discussion and Question and Answer sections were organized as a part of the symposium programme and the contents of a final Symposium Declaration were discussed in detail, but has yet to be published.

## IMPROVING NOMENCLATURAL STABILITY IN MEDICALLY IMPORTANT FUNGI

The pace of name changes in medically important fungi has been proving challenging for those working in clinical laboratories or as clinicians dealing with patients. Sixty-seven mycologists working with medically relevant fungi, and representing a wide range of pertinent organizations, have now produced a conceptual framework to help reduce the problem (de Hoog et al. 2023). Amongst other things, the value of using complexes or series names in reporting identifications is recommended when dealing with cryptic species.

The authors further recommend that an online database be maintained and reviewed routinely by a standing committee perhaps convened under ISHAM (International Society for Human and Animal Mycology) to assess the underlying taxonomy of proposals and advise on what changes should be accepted.

As a first step in implementing their proposals, a Table of recommended names to be used for about 250 common medically important fungi is provided, along with names most commonly used in clinical laboratories and synonyms.

## ASIAN MYCOLOGICAL CONGRESS (AMC 2023)

The Asian Mycological Congress, held from 10–13 October 2023 in Busan, Korea, saw close to 500 mycologists coming together. The meeting included three keynote speakers (Wieland Meyer, Irina Druzhinina, and Joseph Heitman), and nine plenary speakers (Dhanunshka Wanasinghe, Daohong Jiang, Yongsun Bahn, Mark Calabon, Geoffrey Gadd, Aaron Mitchell, Akiyoshi Yamada, Jens Frisvad, and John Taylor). The meeting consisted of three concurrent sessions, with one plenary in the morning, followed by another after lunch. There were 115 oral presentations in total, and more than 250 posters.

There were also two workshops organised, one on Mycobiomics, and the other on Freshwater and marine fungi.

The meeting was very well organised by Hyang Burm Lee and his team, with Hyang also providing light musical entertainment during the dinner! For those who missed the meeting, Korea is definitely a place to visit! The next AMA meeting will be in Guangzhou, China in 2025!

## XIX CONGRESS OF EUROPEAN MYCOLOGISTS

The XIX Congress of European mycologists took place in the historic city of Perugia in Italy on 4–8 September 2023.

The meeting was attended by a few hundred mycologists, many of whom were students. The Sunday saw a special workshop on red-listing Mediterranean fungi coordinated by Greg Mueller, followed by an excursion to the historic city centre. Monday saw the official opening of the meeting, followed by the first keynote address by Pedro Crous on the evolution of phytopathogenic fungi. This was followed by symposia on fungal diversity and interactions, fungal omics, and fungal data management and open science.

**Fig. 5:**
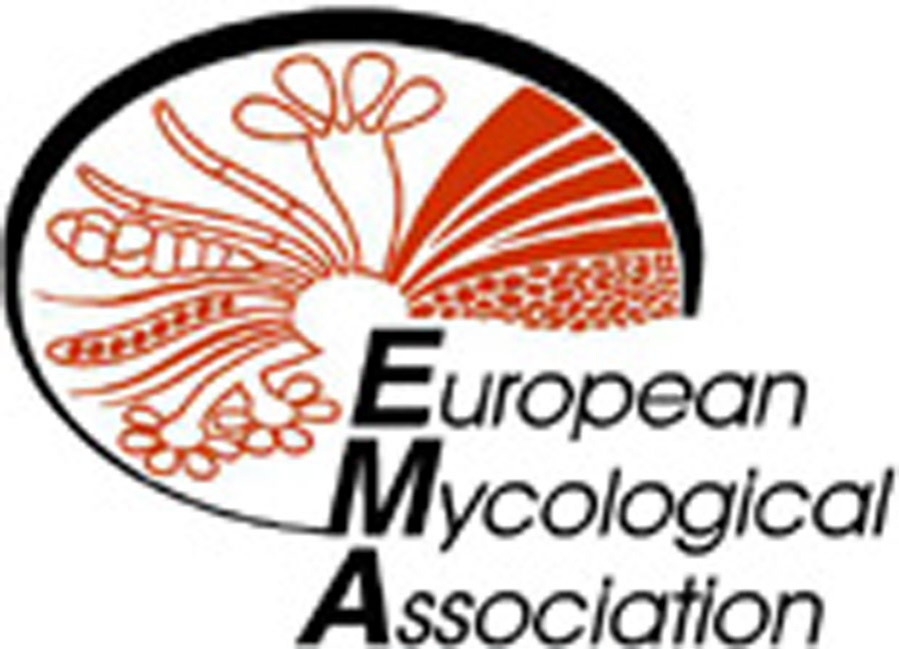
European Mycological Association logo

Tuesday kicked off with a keynote by Lynne Boddy on fungal communities in trees: development in space and time, followed by symposia on fungi in changing ecosystems, fungal conservation, and emerging pathogenic fungi. The day was rounded off with a marvellous dinner of the very best Italian cuisine! Wednesday consisted of two separate excursions, one to Spoleto and the Sacred wood of Monteluco, and the other a visit to the Umbrian truffle ground in the Tiber Valley. Thursday started with a keynote lecture by Giuseppe Venturella, and sessions focussing on medicinal mushrooms, mycology by young scientists (an amazingly exciting symposium!), and was rounded off by the European Mycological Association (EMA; Fig. 5)’s General Assembly. The Friday started with a keynote by Han Wösten on fungal materials, and then sessions on fungi in the bioeconomy and circular economy, and advances in fungal conservation. On the Saturday there was a final post-congress meeting on mycological groups and societies in Europe (*see below*). In all, the XIX congress was hugely successful, and we are looking forward to the next meeting in Estonia in 2027!

Awards made at the General Assembly included: Honorary Membership **(**David W. Minter, UK), Founders' Award (Dmitry Schigel, GBIF, Denmark), Congress Medals (Paola Bonfante, Italy and Silvano Onofri, Italy), Certificates of Excellence Giuseppe Venturella, Italy, Han A. B. Wösten, The Netherlands, and Vincent A. Robert, The Netherlands). The winners of the Mycology by Young Scientists competition were Beniamin Abramczyk, Poland, and Katarzyna Stojek, Poland. There were two Congress Poster competitions: a Jury Prize to Michaela Strmisková, Marek Bárta, Miriam Kadasi Horáková & Katarina Pastirčáková, Slovak Republic, with a second award *ex-æquo* to Miriana Bortolot, Sonia Mazzarino, Alan Pizzinat, Bruno Sacchi, Silvia Perotto, Elena Martino & Alessandra Salvioli di Fossalunga, Italy, and Monika Urbaniak, Elsie Ayamoh Enow, Sylwia Ryszczyńska, Agnieszka Waśkiewicz, Łukasz Stępień, Poland. The winners of the Congress Participants’ Poster Prize were Sylwia Salamon, Polina Havrysh, and Lidia Błaszczyk, Poland.

The EMA Governing Committee elected to serve from 2023 to 2027 was: President Izabela Kałucka (re-elected), Vice-President Irmgard Greilhuber, Secretary Katerina Krusevska, Treasurer Giuseppe Venturella, Membership Secretary Veronica Spinelli, Meetings Secretary Robert Logar, Editor Paulo Oliveira, and Conservation Susana C. Gonçalves).

## REPORT OF A MEETING OF EUROPEAN MYCOLOGICAL GROUPS AND SOCIETIES

This meeting followed that of the XIX Congress of European Mycologists, in Perugia, Italy on 9 August 2023 and aimed first to draw together the current landscape of academic and citizen science-oriented mycological activities in Europe and second to discuss possibilities for collaboration.

More than 55 organizations were represented; the list being shared via the EMA and IMA webpages. During the meeting, 12 talks were presented, covering activities of various mycological groups including the Ukrainian mycological group, Latvian Mycological Society, Polish Mycological Society, Unione Micologica Italiana, British Mycological Society, Estonian Mycological Society, Swedish Mycological Society, Macedonian Mycological Society, Arab Society for Fungal Conservation, a mycological group in Turkey, European Mycological Association, and the International Mycological Association. Possibilities for collaboration and joint activities were then discussed, with the following regarded as of high priority:

- International Fungus Day.
  EMA plans to organize a synchronized International Fungus Day in 2024, as a summary of national fungus weeks and days. The event will also be related to an intensive 24 h long global fungal bioblitz. It should also include a common communication strategy. If you want to join the International Fungus Day working group, contact us. We also encourage you to share information on your national fungus weeks and days with the broader community this year, e.g. on the EMA Facebook page!
- A co-ordinated citizen science campaign to monitor one common and widespread European species.
  This is planned as a rather large action, so more detailed discussion is needed, again if you would like to join the working group on this synchronized citizen science campaign.
- Prepare a list of already existing national fungi recording apps and databases.
  Please contribute to a table being compiled on our website. When completed this will be shared together with a list of the societies.
- A joint project on networking for mycological movements and societies.
  It was agreed to continue with meetings for coordinated European cooperation of mycological movements and societies and we plan to organize regular online meetings. If you would like to coordinate this action don’t hesitate to contact us!

We plan to organize the next in-person meeting on the cooperation of Mycological Groups & Societies during IMC12 in Maastricht. We thank all who participated for this fruitful meeting, and trust it is a good start for further collaborations.

- **Julia Pawłowska, Igor Siedlecki and Izabela Kałucka**
- (julia.z.pawlowska@uw.edu.pl)

## XI LATIN AMERICA CONGRESS OF MYCOLOGY

The congress was held on 7–10 August 2023 in Panama City, Panama. It was preceded by two days of workshops: (1) Introduction of bioinformatic and biostatistics methods for the analysis of fungal communities; (2) The challenge of teaching mycology; (3) Methods to study endophytic and symbiotic fungi; (4) Ectoparasitic fungi associated to insects; (5) Taxonomy, diversity and culturing of entomopathogenic fungi in tropical America, with emphasis on the genus *Cordyceps* s.l.; and (6) Promoting conservation of neotropical fungi through the training of specialists and the extinction rate evaluation of threatened fungi.

The congress was attended by 326 participants from 35 countries. We had eight keynote speakers, 10 symposia, and a total of 318 presentations (oral and posters).

For further information please see https://congresolatinoamericanodemicologia.com/

- **Luis Mejia**
- (LMejia@indicasat.org.pa)

## WESTERDIJK SPRING SYMPOSIUM: FUNGAL EVOLUTION

**Fig. 6:**
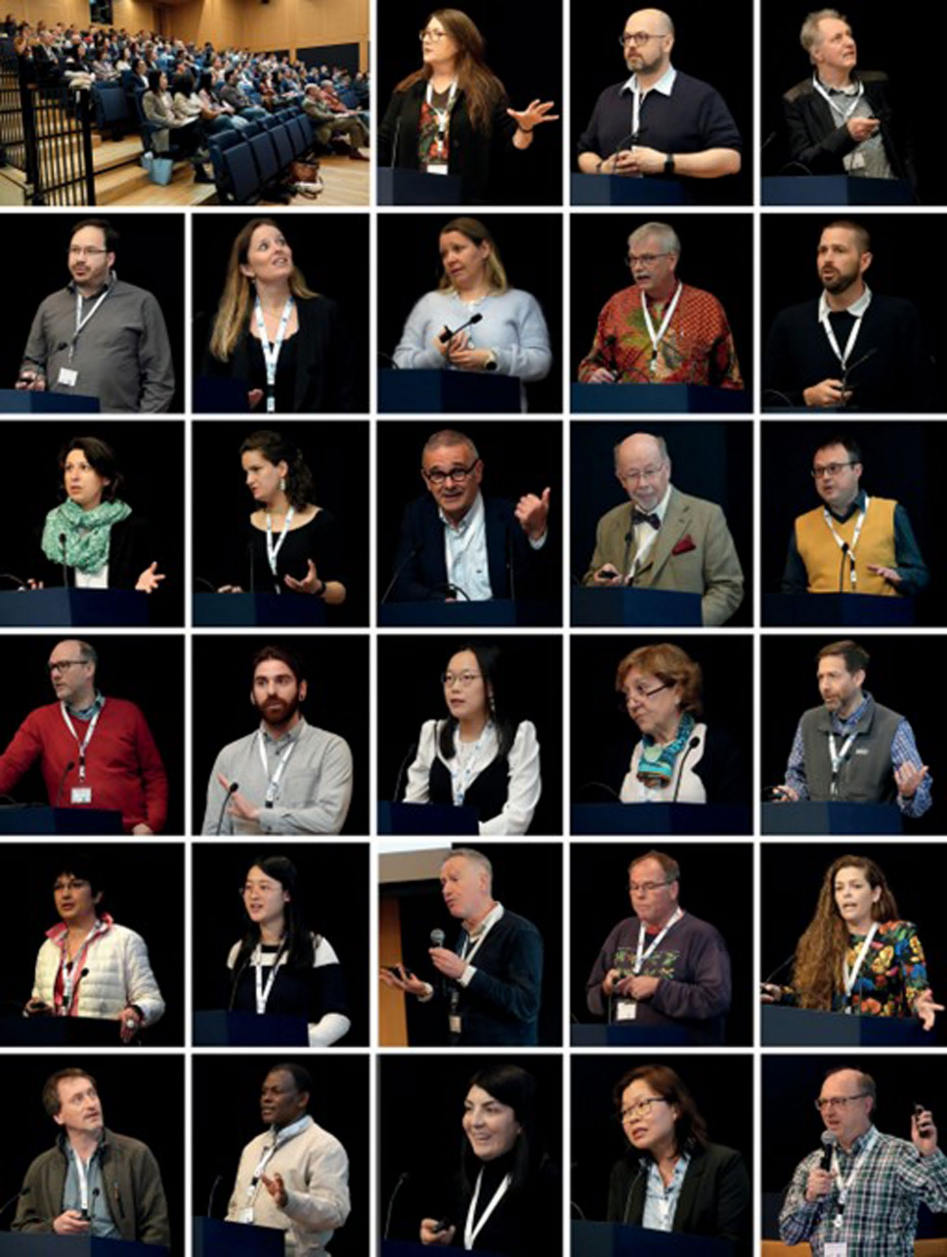
A selection of speakers at the Evolution of Fungi symposium, Amsterdam, 2023

The theme of the 2023 Westerdijk symposium was ‘Fungal Evolution’ and held from 17–18 April 2023 at Trippenhuis, the headquarters of the Royal Dutch Academy of Arts and Sciences in Amsterdam (Fig. 6).

The meeting was attended by 128 participants from 29 nationalities. On the Monday morning, the first session was on ’Do you believe in taxa’ (with Cathie Aime, Michael Seidl, Sybren de Hoog, Marco Thines) which focussed on genome dynamics and evolutionary trends in populations, species and genera, and potential pitfalls in analysing these data. The second session ’Intra- and interspecific variation of food and indoor fungi’ (with Monika Coton, Inger Skrede, Jens Frisvad, and Cobus Visagie) focussed on species boundaries in food and indoor fungi, and their functional diversity. After a light lunch, the third session ‘Microbiota preserved for the future’ (with Tanja Kostic, Isabelle van Thiel, and Vincent Robert) which took a detailed look at biobanking, databanks, tools and resources. Vincent Robert, who was a special speaker, reflected on his past 30 years at the Westerdijk Institute, and future prospects for those working in mycology.

The final session of the day, organized by the International Commission for the Taxonomy of Fungi, was 'von Arx’s dream revisited' where David Hawksworth and Andrey Yurkov presented their views on implementing the use of actively living cultures as types. The session was rounded off by a panel discussion on the topic, drinks, further discussions, poster awards, and then a speakers’ dinner.

The Tuesday started with the announcement of the Johanna Westerdijk Award to Andrey Yurkov, and the Josef Adolf von Arx Award to Cathie Aime (*see below*). The first scientific symposium of the day was on ’Fungal evolution and chemical diversity’ (with Russell Cox, Theo Llewellyn, Xing Zhang, and Olga Genilloud) which focussed on metagenomics and secondary metabolites, followed by ‘Fungal genomics and applications’ (with Scott Baker, Irina Druzhinina, Jiajia Li, and Wilco Meijer) which addressed biotechnology and mycoproteins. The first session after lunch, ‘MycoBiomics’ (with Yasmina Marin-Felix, Miroslav Kolarik, Josphat Matasyoh, Neriman Yilmaz, Jennifer Luangsa-ard, and Markus Gorfer) focussed on secondary metabolites in fungi, and their potential applications. The final session of the day “Human health: fungi on the move” (Eveline Snelders, Vit Hubka, Grit Walther, Auke de Jong) discussed emerging fungal pathogens, azole resistance, and the evolution of pathogenic species complexes. The symposium was then concluded with a discussion and drinks.

- **Pedro W. Crous**
- (p.crous@wi.knaw.nl)

## SOCIEDADE BRASILEIRA DE MICOLOGIA

The Brazilian Society of Mycology (SBMic) began its activities in 1990. Its first congress was in 1995 and its focal point has mostly been its congresses. These have occurred at 3-year intervals, except for the X Congress, which was delayed because of the COVID pandemic and will now take place in February 2024 (https://cbmic2024.com.br/) in Belo Horizonte (Minas Gerais State).

Recent years’ developments have been, among others, the preparation of a website (https://sbmic.org/) and a newsletter (*Boletim Micobiota*). The bulletin first appeared in 2021 and so far has been regularly published quarterly.

The themes explored in the bulletin relate to all aspects of mycology and it intends to communicate not only with the Society members, but also with the Portuguese-speaking public with an interest in fungi as a whole. Therefore, a simple and direct language, combined predominantly with science-based information and attractive images, are preferred over a more formal scientific format.

**Fig. 7:**
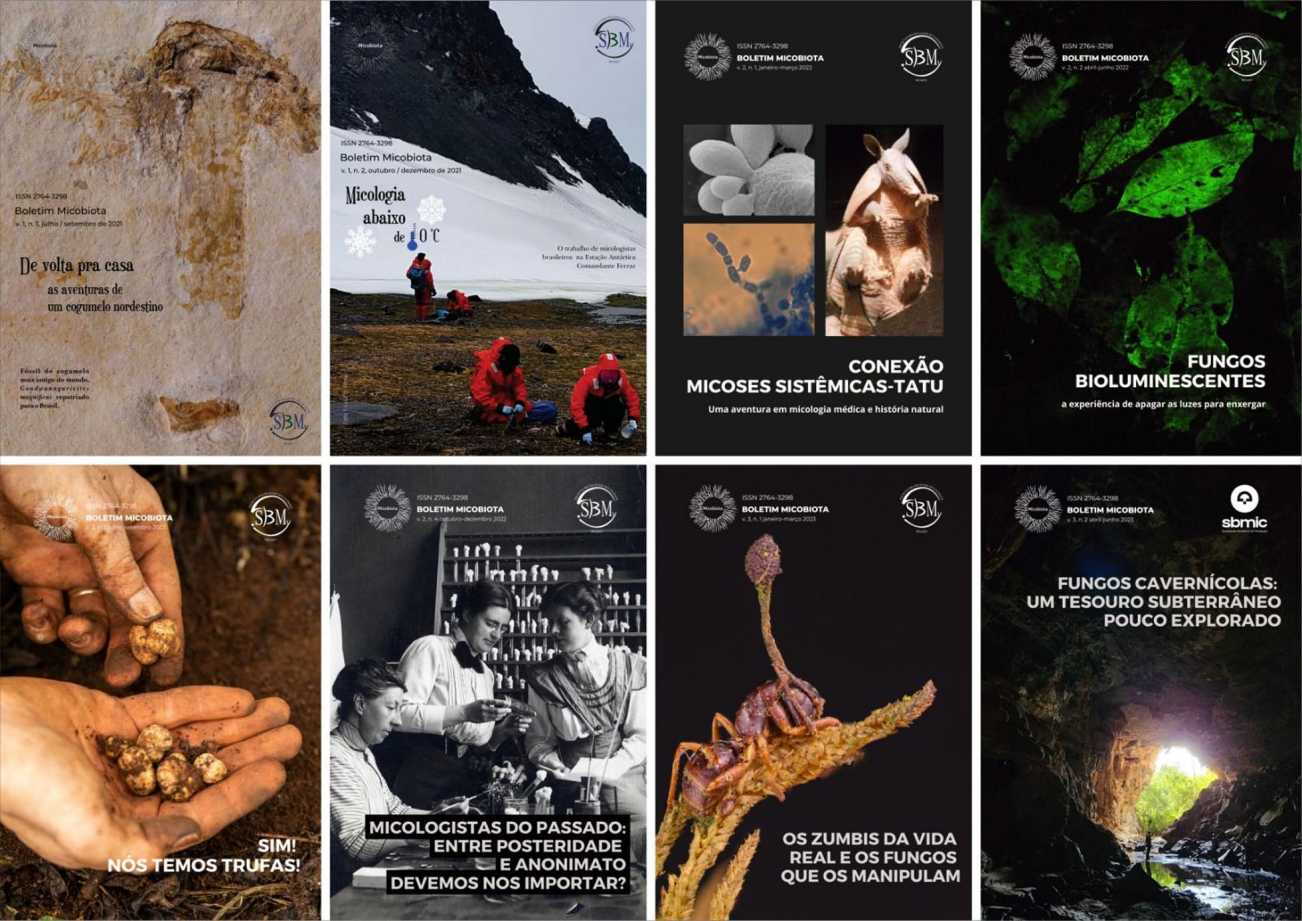
Covers of issues on the Brazilian Mycological Society’s bulletin, *Micobiota*

Over the last editions, a range of subject have featured as front-cover topics (Fig. 7):

- Repatriation of an iconic mushroom fossil (*Gondwanagaricites magnificus*) smuggled out of Brazil.
- Below-zero mycology—on the activities of Brazilian mycologists surveying for fungi in Antarctica.
- Connection between the armadillo and systemic mycoses, based on the seminal research/detective work by the late Bodo Wanke (SBMic founder) and other Brazilian medical mycologists in the Brazilian northeast,
- Bioluminescent fungi in the Amazon forest.
- Discovery of edible truffles in Brazil.
- Mycologists of the past (thoughts on their legacies and institutions).
- Zombie ant-fungi: facts and fiction.

*Mycobiota* is open access, and all numbers and editions are available at https://sbmic.org/micobiotaboletim. Even non-Portuguese speaking myologists find it interesting to browse through the themes and images covered in our bulletin.

- **Eduardo Guatimosim** (*Editor in Chief,* Mycobiota)
- **and Roberto Barreto** (*President, SBMic*)
- (rbarreto@ufv.br)

## 2023 ANNUAL MEETING OF THE MYCOLOGICAL SOCIETY OF CHINA (MSC)

**Fig. 8:**
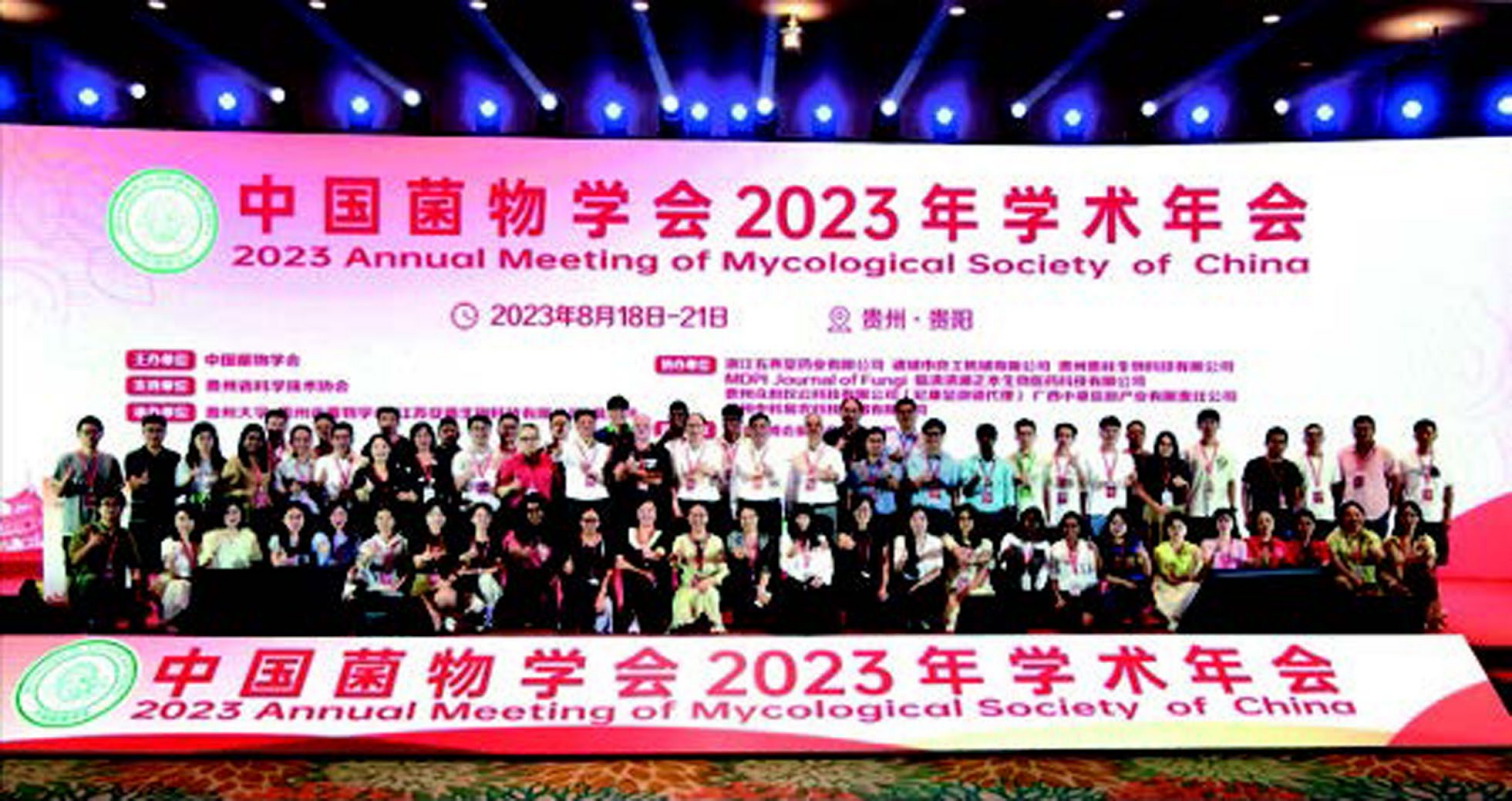
Annual Meeting of the Mycological Society of China, Guiyang, 2023

The Mycological Society of China (MSC) held its annual meeting in Guiyang, Guizhou Province, from 18 to 20 August 2023 (Fig. 8). The event was a tremendous success, attracting over 1200 delegates and commemorating the society’s 30th anniversary since its establishment.

During the opening ceremony, Guo Liangdong, the current president of MSC, provided a historical overview of the society and shared insights into its future development. Keynote lectures were given by Xing-Zhong Liu, Cheng Gao, and Wen-Bing Ying, followed by a plenary by Kevin D. Hyde.

Over the next two days, the meeting featured 290 oral presentations across 11 parallel sections, as well as 42 poster presentations. The event culminated with two plenary lectures delivered by Zheng-Guang Zhang and You-Cai Hu. Additionally, 13 students were recognized and awarded for their outstanding presentations.

The meeting not only highlighted the significant progress made by China in various aspects of mycology but also provided opportunities for networking and enjoyable evenings filled with dinners and the indulgences of Chinese liquor and beers. Overall, it was one of the most successful mycological meetings the MSC has ever held.

## FIFTH IRANIAN MYCOLOGICAL CONGRESS

The fifth Iranian Mycological Congress was held from 26 to 28 August 2023 in Tabriz (East Azerbaijan province, Iran) with the motto “Fungi for better life and safe planet”. The event was organized by the Iranian Mycological Society (IMS) and University of Tabriz, with the support of several Iranian universities, research institutes and private companies.

**Fig. 9:**
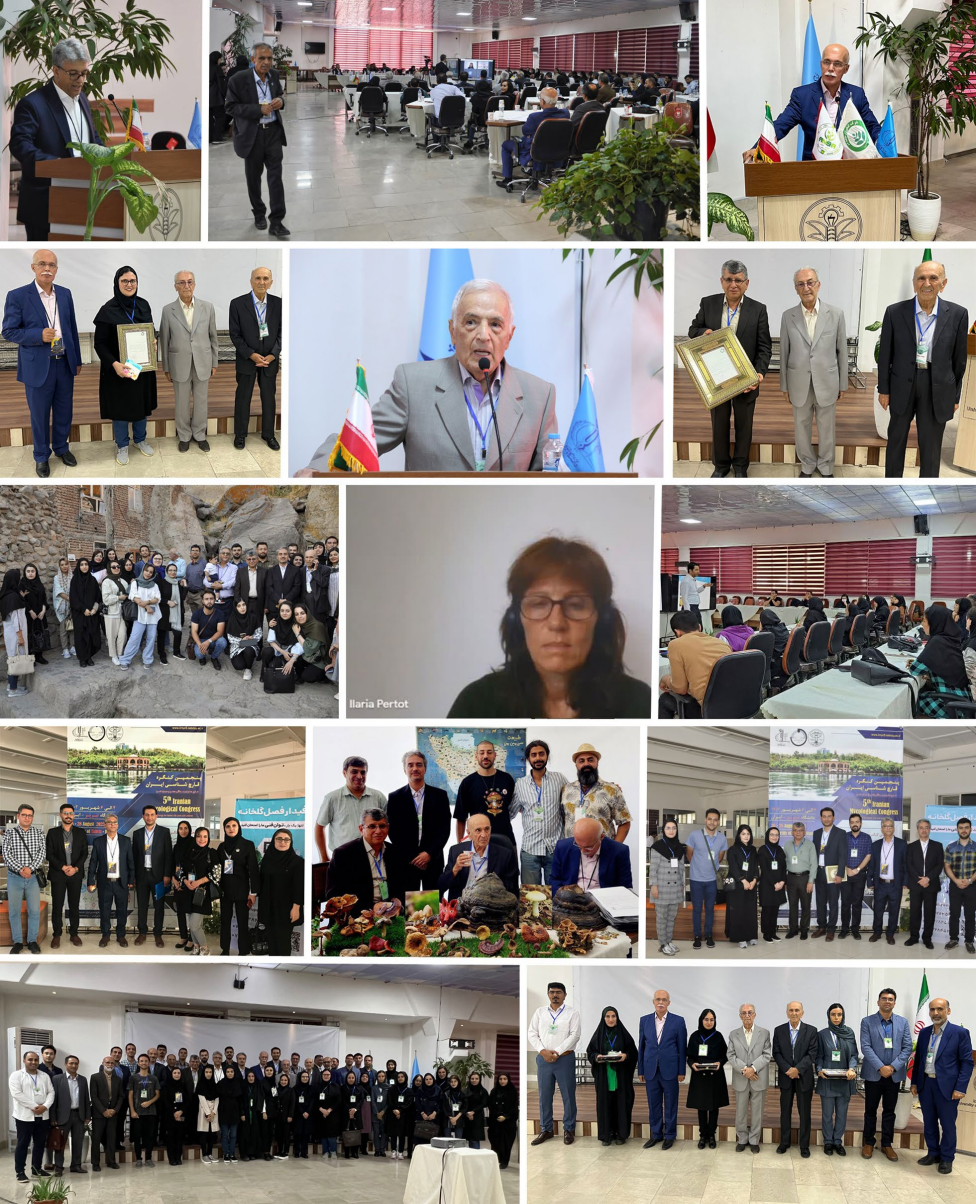
Fifth Iranian Mycological Congress, Tabriz, 2023

The congress attracted more than 175 participants, ranging from 87-year-old retired senior mycologist Djafar Ershad to a 2-year-old-girl, Ania Arzanlou (Fig. 9). It comprised 10 invited international talks, 25 oral presentations, 115 posters, and three workshops. The keynote international lectures were presented by: Ilaria Pertot (Italy), David Hibbett (USA), Eva Stukenbrock (Germany), Jos Houbraken (The Netherlands), Sybren De Hoog (The Netherlands), Mohammad Babadoost (USA), Richard Summerbell (Canada), Allan Patrick Macabeo (The Philippines), Sherif Saeed Ebada Elsayed (Egypt), and Mohammad Bahram (Sweden). There were three post-congress workshops organized, on Principles of Medicinal Mushroom Production, Fungal Secondary Metabolites, and Phylogenetic Analysis.

The 14th General Assembly of IMS was held during the congress, when the 4th Dr Hedjaroud's Award (see *IMA Fungus* **6**(2): (47)–(48), 2016) was presented to Bahram Sharif Nabi of Isfahan University of Technology, and the first Young Mycologist Award was presented to Mounes Bakhshi of the Iranian Research Institute of Plant Protection. In addition, the winners of the 2nd Iranian Mycological Olympiad, an annual academic competition for BSc students, were also given their awards.

A fascinating excursion to Kandovan, known as the only “inhabited rocky village” in the world and one of the most famous and astonishing natural and cultural attractions of East Azarbaijan, was also organized for the delegates.

During the congress a fantastic fungal photography exhibition was also held and displayed images taken by congress participants featuring macro- and microscopic fungi of Iran.

The congress closed with reports from the Society’s President (Mohammad Javan Nikkhah), Secretary (Mahdi Arzanlou), and Executive Manager (Reza Farshbaf). Awards were also given then for the best oral and poster presentations as well as the best photographs from the photography exhibition.

- **Mahdi Arzanlou and Mounes Bakhshi**
- (mounesbakhshi@gmail.com)

## AWARDS

### IMA AWARDS 2024

To acknowledge the various contributions of its members, the IMA will be making three awards in the following categories at IMC12:

- **De Bary Medal**—Based on outstanding career research.
- **Ainsworth Medal**—For recognition of extraordinary service to world mycology.
- **Young Mycologist Award**—Awarded to outstanding mycologists early in their career.

The IMA Awards Committee for 2024 consists of the Council Members, and up to three members from the Executive Committee (EC). Chiharu Nakashima, head of the IMA Awards committee, will accept nominations for the Ainsworth Medal and the De Bary Medal. The nominations for the Young Mycologist Awards should be addressed to the Regional Mycological Member Organizations (RMMOs) of the region in which the Nominee achieved his research results. Contact details for the RMMOs are available on the IMA website.

The deadline for applications is **15 February 2024**, to coincide with the IMC 12 meeting in Maastricht, The Netherlands the same year.

General Requirements for application of the awards are:

- An individual may receive the same IMA Award only once.
- Self-nomination is not allowed.
- Nominators must be members of the IMA.
- Nominees who are not chosen for the prize may be re-nominated for up to two additional terms (within the year limit linked to the specific award).

Documents to be submitted should include a nominating letter, including a detailed evaluation of the nominee’s contributions to Mycology, and a current *curriculum vitae*. Nominations should be sent to the head of the IMA Awards Committee for the Ainsworth Medal and De Bary Medal, while those for the Young Mycologist Award should be sent to the respective RMMO representative.

The awards consist of a certificate. Note that the Committee may not grant an award in a given year if there is no suitable candidate that meets the criteria. Presentation of the awards will take place at the awards ceremony at IMC 12 in 2024.

Contact Chiharu Nakashima (Japan), Vice-president IMA Awards (IMA Executive Member seconded to the IMA Council) to whom applications should be submitted and who can provide further information if required. Email: chiharu@bio.mie-u.ac.jp.

### THE FUTURE IS FUNGI AWARD

**Fig. 10:**
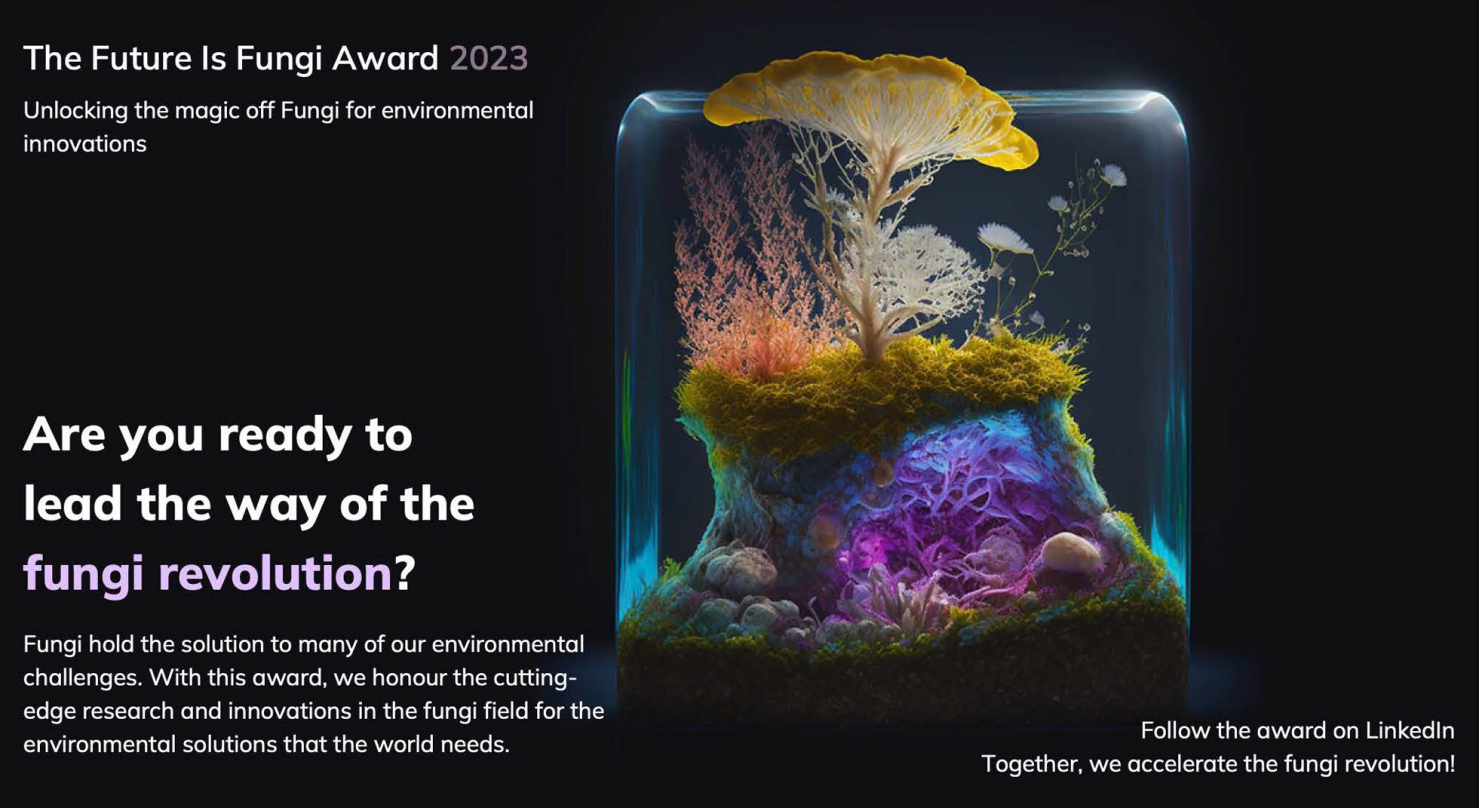
The Future is Fungi Award

The Future is Fungi Award (Fig. 10) is a new venture that honours frontier and cutting-edge research and start-up innovation leveraging fungi for environmental solutions that the world needs. This award aims to honour frontier fungal research, inspire more research and innovation leveraging fungi for environmental solutions, nudge commercialization of research into real-world solutions and to tell the broader world about the potential of fungi for environmental solutions.

The research winner for the 2023 award was Jens Laurids Sørensen from Aalborg University. He won this for his research on utilizing fungi as a bio-battery for more sustainable storage of energy. He has a proof of concept and a prototype in place, and his solution provides a truly groundbreaking way of utilizing fungi for more sustainable storage of energy and inspiring others to open their minds to the possibilities of fungi.

o The start-ups Novobiom and MycoMine share the start-up 1st prize; both show how we can harness fungi to clean up contaminated soil and water through mycoremediation. In addition, Novobiom also converts textile waste into new bioactive compounds, with the use of fungi, showing the way for more bio-based solutions and circularity.
o In addition to receiving € 10,000, the winners each received mentorship and access to a broad network.
o The Award looks forward to honouring more fungal-focused researchers with pioneering solutions on how we can harness fungi to put our common globe on a more sustainable trajectory. We look forward to announcing the application period for 2024.

You can read more about the award on: https://www.futureisfungi.org/, and see the Award Ceremony for 2023 on: https://www.futureisfungi.org/award-ceremony

and follow the award on LinkedIn: https://www.linkedin.com/company/the-future-is-fungi-award/

- **Susanne Gløersen**
- *Initiator of The Future is Fungi Award*
- (suSanne@thefuturesifungi.org)

### ABDEL-AZEEM AND STAMETS’ AWARD FOR RECORDING BLUING *PSILOCYBE* IN AFRICA

**Fig. 11:**
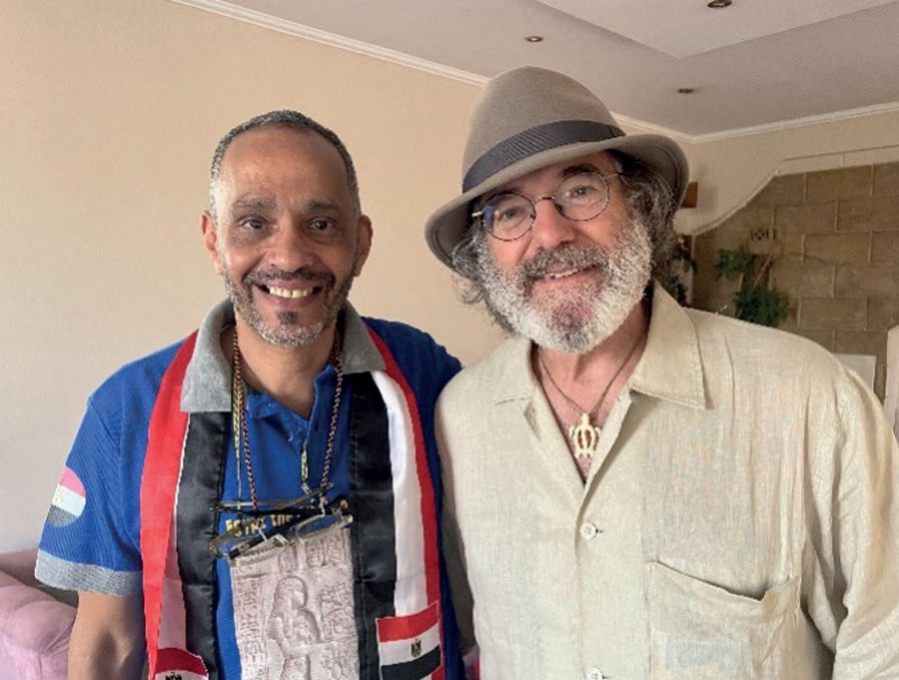
Abdel-Azeem and Paul Stamets

The Arab Society for Fungal Conservation (ASFC) and African Mycological Association (AfriMA) are honoured to announce the Abdel-Azeem and Stamets’ Award (Fig. 11) for recording psilocybin-active *Psilocybe* species in Africa-2023. More than 140 species of mushrooms are known to be psilocybin active, with likely more to be discovered, especially in Africa. *Psilocybe* is the best-known genus for psilocybin containing mushrooms and these are regularly found in substrates such as soil, dung, wood, and mosses.

Psilocybin-active psilocybes are under-reported in Africa despite the likelihood that *Psilocybe cubensis*, the species most commonly cultivated and used throughout the world, originated on this continent.

In particular, *Psilocybe* species are thought to have once been native to the Nile River regions, in southern Algeria, and elsewhere in Africa, before desertification created adverse conditions. Hence, the goal of this award is to record and collect these now rare species.

To date, studies have shown that psilocybin therapy is beneficial in relieving symptoms of treatment-resistant depression, obsessive compulsive disorder, and other mental health disorders. Psilocybin has also shown effectiveness at easing fear and anxiety in people with terminal cancer.

The aim of this new Award is to document and update information related to the biodiversity and conservation of this genus in Africa.

The competition runs to the end of July 2024, and any mycologist in Africa interested in recording bluing *Psilocybe* (including GPS, field photos, spore prints and living culture) can contribute. The Award is for US $ 500, for the first position, 300 for the second, and 200 for the third. All recorded data (GPS, photographs, spore prints, pure culture, etc.) should be sent to award@fungiofegypt.com along with any questions regarding regulations related to the delivery of living cultures.

- **Ahmed Ismail**
- (ma.ah.ismail@gmail.com)

## WI-KNAW FUNGAL BIODIVERSITY INSTITUTE AWARDS 2023

Two awards were made by the Westerdijk Institute at the FungaL Evolution symposium in Amsterdam on 18 April 2023.

### JOHANNA WESTERDIJK AWARD: ANDREY YURKOV

*Awarded on special occasions to an individual who has made an outstanding contribution to the culture collection of the CBS Fungal Biodiversity Centre, marking a distinguished career in mycology. Nominees for the award will be evaluated on the basis of quality, originality, and quantity of their contributions to the collection, and on the basis of associated mycological research in general.*

Andrey Yurkov is one of the World’s leading authorities in yeast taxonomy, with particular expertise on the systematics, biodiversity, and evolution of yeasts. He is a sought-after speaker and organizer and is actively involved as a reviewer for numerous journals, and serves on committees in the World Federation for Culture Collections (WFCC), the IMA, the Yeast Foundation and, theyeasts.org.

He is recognized as a special recipient of the Westerdijk Award today, however, as he has deposited a huge collection of yeasts in the culture collection, thereby ensuring that these fungi remain available for research by future generations. These cultures were collected by him and his team over many years, and as such represent a major investment of time and resources. As a mycological community, we thus thank him for this incredible foundation, and trust that students in years to come will continue to build on this wonderful platform.

### JOSEF ADOLF VON ARX AWARD: MARY CATHERINE ("CATHIE") AIME

*Awarded on special occasions to an individual who has made an outstanding contribution to taxonomic research of fungal biodiversity, marking a distinguished career in mycology. Nominees for the award will be evaluated on the basis of quality, originality, and quantity of their contributions in the field of fungal taxonomy.*

Cathie Aime obtained her PhD at the Virginia Polytechnic Institute and State University in 2001. She has been Curator of the US National Fungus Collections in Beltsville, a Professor at Louisiana State University, and is presently Professor at the Department Botany & Plant Pathology, Purdue University, where she is also Director of the Arthur & Kriebel Herbaria. Throughout her career she has received numerous awards, and is also a Fellow of the Mycological Society of America, and the Linnean Society of London.

Cathie’s research emphasis has focussed on two areas, the rust fungi *Pucciniomycotina* and biodiversity. Cathie has published extensively in a wide range of journals, and her papers are extremely well cited, appreciated, and used by the community. It thus gives us great pleasure to award Cathie Aime the Josef Adolf von Arx award for fungal systematic research.

- **Pedro W. Crous**
- (p.crous@wi.knaw.nl)

## IN MEMORIAM

## Lorelei Louise Lehwalder Norvell (1943–2023)

**Fig. 12:**
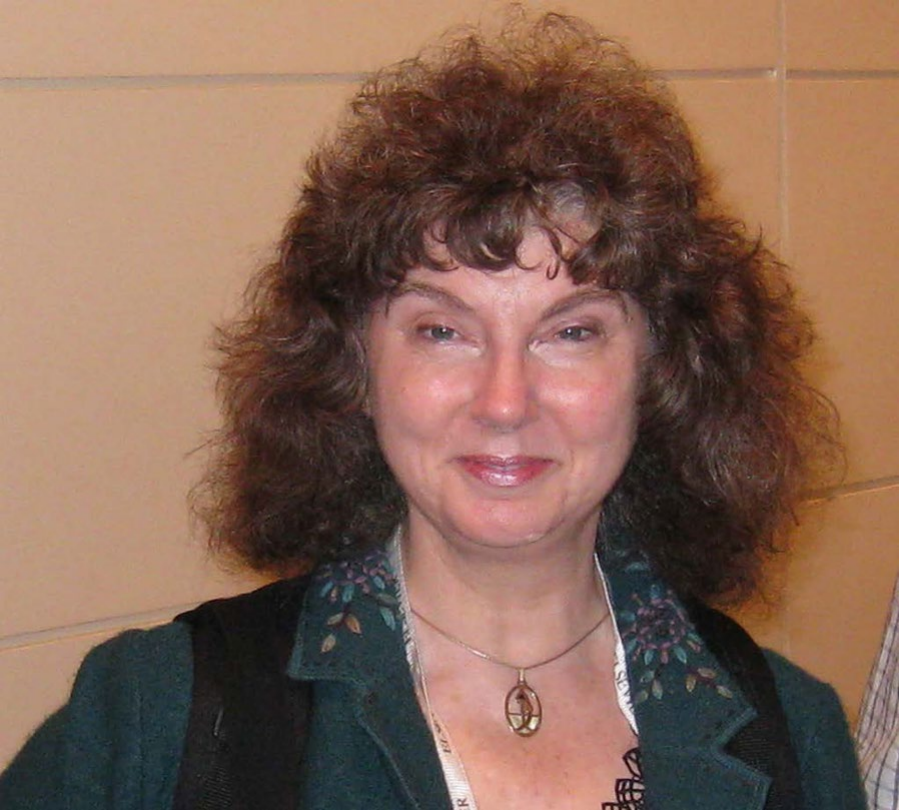
Lorelei Norvell (1943–2023) at IMC10 in Edinburgh in 2010

Lorelei Norvell (Fig. 12), an active member of the International Botanical Congress’ Nomenclature Committee for Fungi since 1999 and its secretary 2008–2013, passed away on 4 August 2023, still diligently working as Editor-in-Chief of *Mycotaxon*, a role she had since 2005. Unlike many IMA members, Lorelei did not begin her mycological career studying fungi, biology, or even science. In college she infamously blew up a chemistry lab, and instead focussed on languages. She studied German for her AB (Knox College 1964) followed by obtaining a high school teacher accreditation (Southern Illinois University, Edwardsville) while her husband Todd served in Vietnam. Next, she studied Slavic languages (Russian, Serbo-Croatian, etc.) for her MA (University of Texas, Austin 1969) while Todd studied law. She followed the careers and frequent moves of her chemist father, David Lehwalder and wife Betty, and then the career of her husband Todd, whom she had married in 1964. Once settled in Portland, Oregon, she began a celebrated leaded glass artist studio while raising her two sons, Forrest and Owen in their designer home in a lush west coast rainforest.

A nature lover since childhood, Lorelei identified plants and animals surrounding their home with nature guides, but ran up against difficulty with mushrooms! She loved an intellectual challenge, especially involving nature, and joined the Oregon Mycological Society (OMS), soon becoming its president, curator of its fungarium, and one of the most knowledgeable amateurs in the Pacific Northwest. She developed a database on all Pacific North-West (PNW) mushrooms and spearheaded the OMS ten-year-long chanterelle harvesting effect study on Mt Hood by a dedicated OMS field crew; that is now cited as a standard work. In 1983 she also began writing humorous educational articles in *Mushroom*, a journal illustrated by doe-eyed mushroom characters, which continued for 14 years. Lorelei took part in many forays by OMS and the PNW Key Council, meeting famous mycologists such as Daniel Stuntz, Nancy Weber Smith, Roy Watling, and Joe Ammirati. She aspired to become a mycologist, but first had to obtain a degree in science to enter a doctorate program. She promptly enrolled in Portland State University which granted her a BSc in botany in 1990, and then she entered a PhD program at the University of Washington as a student of Joseph F. Ammirati. Lorelei’s study focussed on the agaric genus *Phaeocollybia,* in part because the PNW is a hot spot of diversity for the genus and because Joe Ammirati and his professor were familiar with the genus, and she had also met Scott Redhead who was interested in *Phaeocollybia* from adjacent British Columbia. Field trips in the old growth forests of the Olympic Peninsula Mountains and the Carmanah Valley on Vancouver Island, and a two month-long survey in 1992 ranging from British Columbia to California with George Barron and Scott set the stage. Joe ensured that she adopt the latest technology based on DNA analyses. Ultimately Lorelei graduate with her PhD in 1998, overlapping residency with fellow students, Brandon Matheny, Sharmin Gamiet, and Katie Glew. During that time she served as an instructor at the off the grid Wild Mushroom Conference at Breitenbush, Oregon, in 1991, 1996 and 1997 and was one of the principle PNW Key Council members, and much loved by senior OMS members, especially Maggie Rogers, Judy Roger, and Catherine “Kit” Scates, and also west coast notables such as Paul Stamets, David Arora, Dennis Desjardin, Jim Trappe, and David Largent, to name a few.

Lorelei was awarded a Mycological Society of America (MSA) Graduate Fellowship Award in 1993 and a Fellowship by the Society in 2005. She soon became the world expert on *Phaeocollybia*, corresponding with Egon Horak and travelling with Roy Halling to Costa Rica to study them. Twelve new *Phaeocollybia* species were described by her along with a PNW *Cantharellus* and *Hydnum*, and also the genus *Chromosera*. She began her private consulting company, Pacific Northwest Mycology Service, to conduct surveys and then joined forces with Ron Exeter to study and publish manuals on *Phaeocollybia, Ramaria,* and chanterelles. She also joined forces with Ron Petersen and Karen Hughes, becoming a All-Taxa (fungal) Biological Inventory coordinator for the Great Smoky Mountain National Park in 1999–2000.

She was editor of the MSA newsletter *Inoculum* from 1998–2002 and then served as secretary for MSA for 2000–2004. Lorelei assisted with the International Botanical Congress in Melbourne, Australia, in 2011, often meeting with David Hawksworth, Tom May and Shaun Pennycook there and at other congresses to progress nomenclatural issues.

Lorelei led a truly remarkable, energetic, and happy life. She twice beat cancer to fulfil a wonderful legacy, and then it took her. The generic name *Loreleia* was published in 2002 in honour of her contributions that would continue another 21 years.

A fuller account of Lorelei’s life and achievements is provided by Redhead et al. (2023), and a tribute to her enormous contribution to *Mycotaxon* is paid by Noni Korf (2023), eldest daughter of the journal’s founder Richard P. Korf and subsequently its manager, who announced the ending of the publication of the journal.

- **Scott A. Redhead and Joseph F. Ammirati**
- (scott.redhead@AGR.GC.CA)

## Takashi Matsushima (1930–2023)

**Fig. 13:**
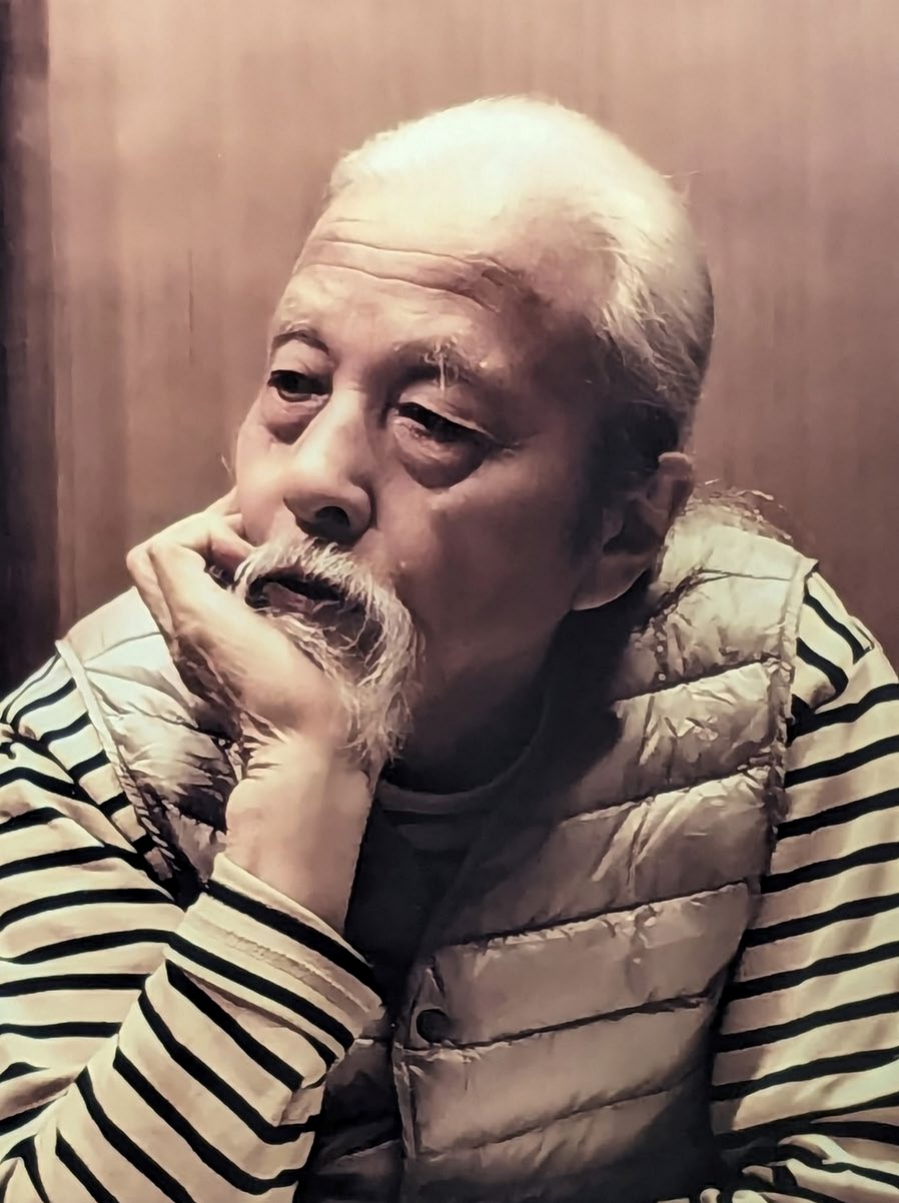
Takashi Matsushima (1930–2023) at home in 2018

We are sorry to announce the death of Takashi Matsushima (Fig. 13) on 15 August 2023 in Kobe, Japan, at the age of 92, after treatment for biliary tract cancer and acute pancreatitis. He was one of the most famous Japanese mycologists, who described many hyphomycetes and other fungi with unparalleled line drawings and photographs for many years (e.g. Matsushima 1971, 1975). In honour of his enormous contributions to hyphomycete taxonomy, many new fungal taxa were dedicated to him: *Matsushimaea* Subram. 1978, *Matsushimamyces* Rah. Sharma & Roh. Sharma 2015, *Matsushimiella* R.F. Castañeda & Heredia 2001, *Matsushimomyces* V.G. Rao & Varghese 1979, *Chloridium matsushimae* W. Gams & Hol.-Jech. 1976, *Didymostilbe matsushimae* Seifert 1985, and *Sporidesmium matsushimae* S. Hughes 1979, among others.

He started working on taxonomy of fungi at the National Institute of Hygienic Health Sciences, Tokyo in 1952, moved to The University of Tokyo in 1962, to Shionogi (Osaka) in 1964, and then founded the Matsushima Fungus Collection (MFC) in Kobe in 1971. The MFC was partly supported by Shionogi. Type materials (slide preparations, dried cultures, and the original figure plates) and living cultures were respectively preserved in MFC and Shionogi, but many were severely damaged or destroyed by the Great Hanshin Earthquake (GHE) in Kobe in 1995. Keisuke Matsushima, eldest son of T. Matsushima, later joined MFC and Shionogi to support Takishi’s studies, and together they co-authored two publications.

To collect microfungi, T. Matsushima visited many places in the world (e.g., South Pacific Ocean, Papua-New Guinea, Australia, Malaysia, Borneo, China, Taiwan, India, South Africa, Peru, Cuba, Alaska), and he interacted with several mycologists at home and abroad (e.g., Y. Kobayasi, K. Tubaki, H. Kurata, T.R. Nag Raj, A. Nawawi, R.H. Petersen, J.W. Carmichael). After the GHE, Petersen was kind enough to help him during a visit to the United States.

The contact address for MFC is: Keisuke Matsushima, 3–2-5 Mikage, Higashinada-ku, Kobe, Hyogo 658–0047, Japan (kineosporia@gmail.com) but sadly most of the holotype specimens and ex-type cultures are no longer extant.

- **Gen Okada and Keisuke Matsushima**
- (gokada@a.riken.jp)

[We were also sad to learn from the pages of *Inoculum* of the passing of **Irma Josefa Gamundi** (1927–2023) and **Martha Jane Powell** (1948–2023), but this news reached us too late to be able to organize tributes in this issue of *MycoNews*.]

## BIRTHDAY GREETINGS

### Bryce Kendrick at 90

**Fig. 14:**
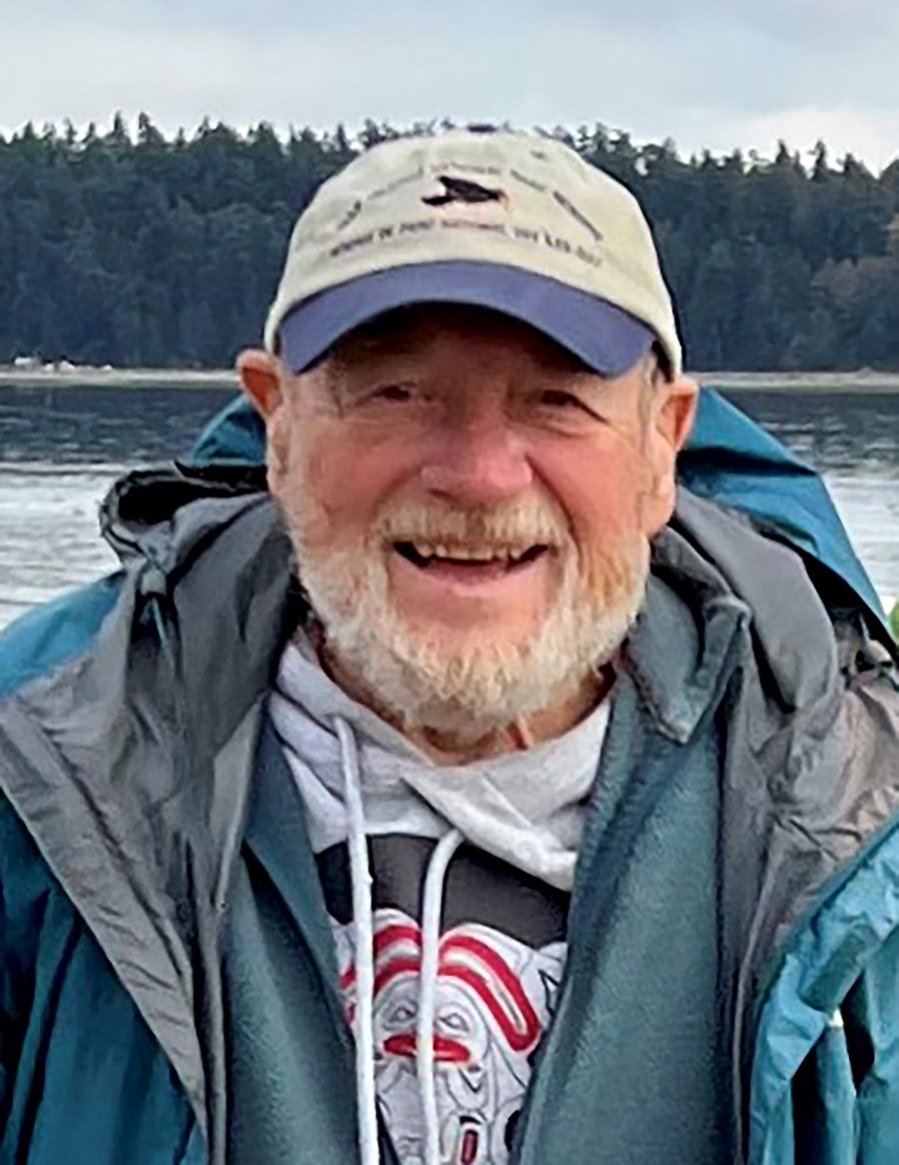
Bryce Kendrick

Bryce Kendrick (Fig. 14) celebrated his 90th birthday on 3 December 2023.

Bryce’s 9.30 lectures at the University of Waterloo were an undergraduate highlight for many students, like a jolt of caffeine after the 8.30 zoology snooze fest. He cracked jokes, unveiled his vast collection of 35 mm slides that portrayed strange, microscopic organisms, and told us stories about his research on hyphomycetes. Biology came alive. To shelter his students from the exorbitant prices of commercial textbooks, he wrote his own, a cerlox-bound book that he sold at cost. First published in 1985, this eventually morphed into his popular (but now commercially published) textbook T*he Fifth Kingdom* (Kendrick 2017), of which a fifth version is now in preparation.

His grad students and post docs worked on whatever aroused their passions, and Bryce tried to keep up. They were a diverse, charismatic crew. In my time, they included MSc student Shannon Berch (studying arbuscular mycorrhizae; my ever-patient Teaching Assistant in introductory mycology), PhD student Frank DiCosmo (who laughed with delight at every surprise the coelomycetes threw at him), a Greek PhD student John Michaelides (who showed us air-bubble-trapping three dimensional helical conidia floating in local rain-filled ponds), and the introverted but passionate perpetual post-doc T.R. Nag Raj (known to all as Nag, who generally only spoke about the appenDAges of his beloved coelos).

After a touch of burnout following a term as Dean of Graduate Studies, Bryce took an early retirement and migrated with his wife Laurie to Vancouver Island. His plan was to reawaken and re-explore his interest in marine biology, which he did while entertaining his granddaughters with his erudite Liverpudlian accent. But he also became involved in Green Politics, and remains an active environmentalist, toiling to extirpate invasive plants from regional shorelines. All the while, he taught correspondence courses, consulted on mouldy buildings, and continues as an adjunct professor at the University of Victoria. Bryce has lost none of his inspirational magic. Last year, he proudly shared a stack of appreciative letters with me that he’d received from undergrad students of a recent field course at the Bamfield Marine Sciences Centre.

On behalf of the large retinue of students and colleagues of several generations, and of mycologists everywhere, we wish Bryce the happiest of 90th birthdays, with many more to follow.

- **Keith A. Seifert**
- (stilbella@hotmail.com)

[See also “Happy birthday Bryce!” published on the occasion of Bryce’s 80th birthday in *IMA Fungus* **4**: (62), 2013.]

### Maria Ławrynowicz

**Fig. 15:**
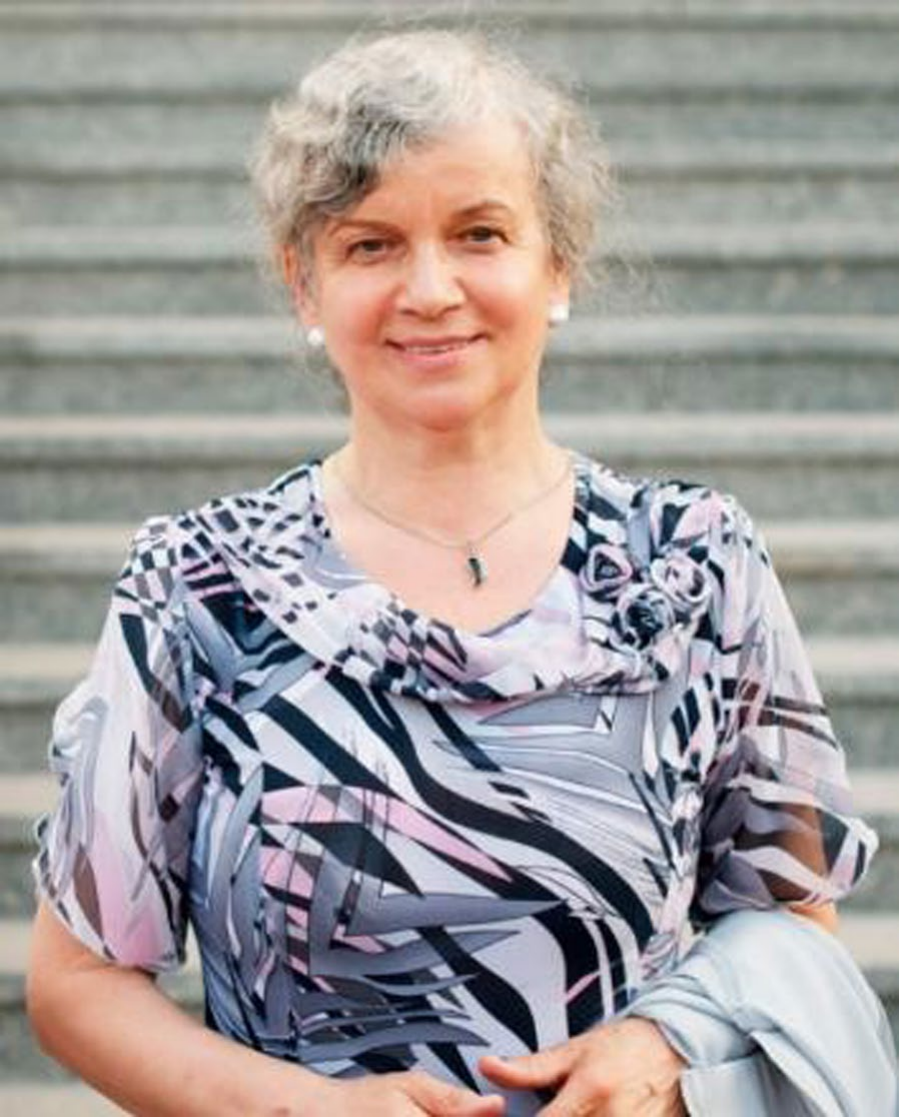
Maria Ławrynowicz

Maria Ławrynowicz (Fig. 15) celebrated her 80th birthday on 17 May 2023. Born in Mstów (Silesian Voivodeship, Poland), since her studies she has been associated with the University of Łódź and worked there for 48 years. She obtained an MSc in 1966 and defended her PhD thesis on the diversity of macroscopic fungi in nature reserves of Central Poland in 1971. Encouraged by Alina Skirgiełło, she focused her scientific career on hypogeous fungi and in 1984 received her DSc (habilitation) for the study of taxonomy and distribution of hypogeous ascomycetes in Poland. She received the title of full professor in 1994. While building up the mycological team at the University of Łódź, she subsequently headed the Mycology Laboratory (1986–1993), Department of Mycology (1993–2000), and Department of Algology and Mycology (2000–2013) until her retirement).

As author and co-author, she published more than 200 scientific papers, chapters and books on fungal diversity, taxonomy, coenology, and conservation. From the very early stages of her career, she also focused on building up the Herbarium Universitatis Lodziensis collection of fungi (Fungarium) which currently contains nearly 60,000 specimens. Maria supervised 87 master's students and 10 PhD students. For 20 years, she directed doctoral studies in ecology and environmental protection at the University of Łódź. During that time, 136 PhD candidates completed this programme.

Maria strongly engaged in nature protection and made a large contribution to the protection of fungi in Poland. Being the representative of the Mycological Section of the Polish Botanical Society, she presented a proposal for the legal protection of mushrooms, enclosing a list of 23 species. The proposal was accepted, and the list of fungi was included in the species protection law in 1983—for the first time in the world. She went on to coauthor red lists of fungi in Poland in 1986, 1992, and 2006. Since 2004, she has been a member of the State Council for Nature Protection. For many years she represented Poland in the European Council for the Conservation of Fungi and was its Chair in 1995–1999. She is also a member of the IUCN SSC Fungal Conservation Committee.

For many years Maria was a member of the Nature Conservation Committee and the Botany Committee of the Polish Academy of Sciences. She has been a member of the Polish Botanical Society since 1966 and was its Secretary-General in 1989–1992. Also, she was a *spiritus movens* and one of the key initiators of the foundation of the Polish Mycological Society in 2012. Since 1966, she has participated in Congresses of European Mycologists building up the link between the Polish and international mycological communities; in 2019 she was presented with the European Mycological Association’s Congress Medal.

She is an honorary member of the Polish Botanical Society, Polish Mycological Society, and European Mycological Association. She was honoured with the Knight's Cross of the Order of Polonia Restituta in 1997, and the Medal of the National Education Commission in 2006.

We thank Maria Ławrynowicz for her lifetime achievements in mycology and for her inspiration and care over several generations of Polish mycologists. We send our sincere congratulations and best wishes on the occasion of this special birthday on behalf of all Polish Mycologists, Collaborators, and Friends.

- **Julia Pawłowska, Izabela Kałucka,**
- **Małgorzata Ruszkiewicz-Michalska,**
- **and Dominika Ślusarczyk**
- (julia.z.pawlowska@uw.edu.pl)

### Yu Li

**Fig. 16:**
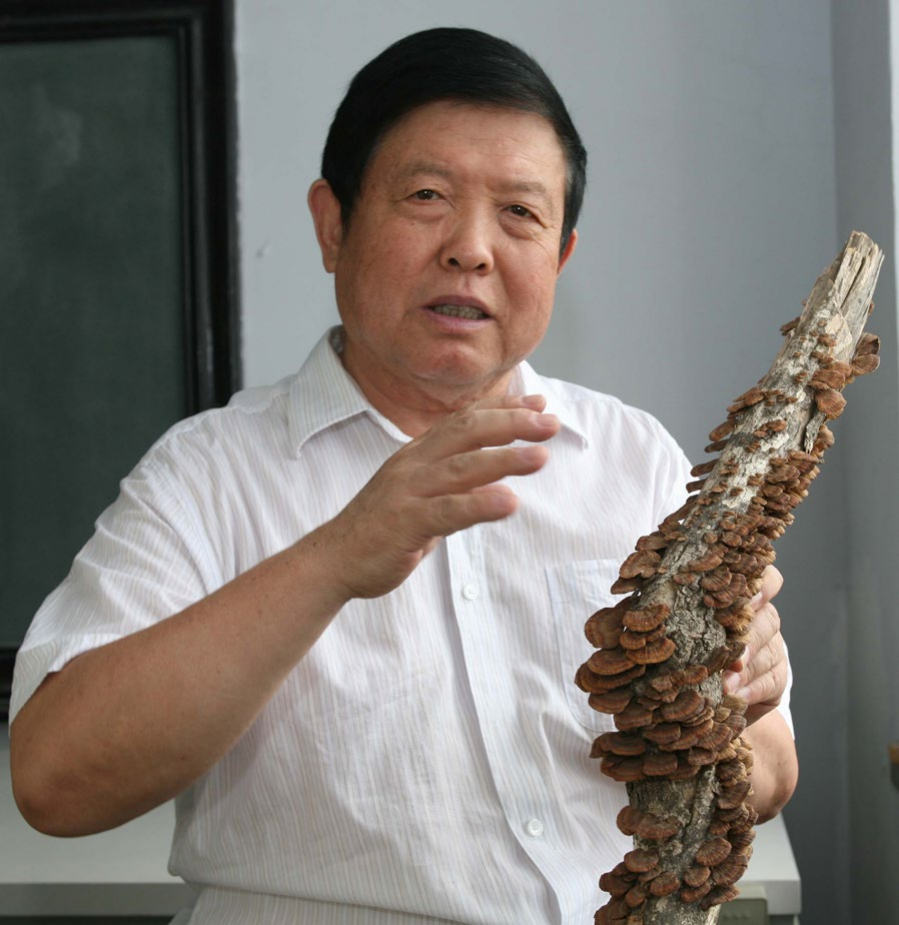
Yu Li

Chinese mycologist Li yu (Fig. 16), now in his 80th year, was born in Jinan, Shandong Province, China. He graduated with the degrees of Master of Science of the Chinese Academy of Sciences, and Doctor of Agriculture of Tsukuba University, Japan. He is an Academician of the Chinese Academy of Engineering, Foreign Academician of the Russian Academy of Sciences, Chairman of International Society for Medicinal Mushrooms, professor and doctoral supervisor at Jilin Agricultural University, recognized as a National Excellent Educator and Science and Technology Worker, and received the Tai Fanglan Distinguished Achievement Award.

In the past 50 years, Li yu has devoted himself to research in mycology and particularly the engineering and industrialization of edible fungi. He successfully combined basic theory with applied technology, relying on innovative achievements to solve technological problems of edible fungi engineering in northern China, thereby promoting the advancement of the industry.

He established a Fungus GenBank that ranks highly in China, including more than 400 species of myxomycetes, around two thirds of the world’s known species. Until now, he has reported 46 new species of myxomycete species and his *Systematic studies on representative taxa in Myxomycetes* was awarded Second Prize for National Natural Science in China.

His *Fungal diversity protection innovation system* earned him first prize as an outstanding scientific and technology progress award of the access to the Ministry of education. He has screened and cultivated 50 species of edible fungi, and improved creatively eight key skills, resulting in almost six billion RMB of direct economic value to China. *Breeding Selection of New Edible Fungi Species, Research on the Concerned Skill for High Harvest and Establishment of Industrializing Creative System* was awarded first prize of the Jilin Provincial Advance of Science and Technology, while his *New Cultivation Mode for High Production and Efficiency of Northern Edible Fungi* achieved second prize of the Jilin Provincial Advance of Science and Technology. *Medicinal fungus resources and its development and utilization* was awarded first prize for scientific and technological progress in Jilin Province.

He was so far published more than 600 academic papers, 25 books, and authored 20 patents. Li yu founded the *Journal of Fungal Research*, and established the Engineering Research Center of Chinese Ministry of Education for Edible and Medicinal Fungi in Jilin. He is one of founders who established a comprehensive system of Mycology and Edible Fungi Engineering faculties in China which train junior college, undergraduate, master, doctoral, and post-doctoral students. His team was recognized as a National Educational Team, and, leading his team, he took charge of more than 50 national or provincial grants on fungal science, technology and engineering. As a President, Li yu hosted six international and cross-strait (i.e. including Taiwan) academic conferences. Li yu’s contribution towards Fungi is extraordinary not only in China but for the world of commercial mushroom production.

Two special issues have marked this important birthday, one in *Mycosphere* prefaced by a tribute from Hyde et al. (2023), and the other in the *Journal of Fungal Research* with several papers honouring his work (e.g. Changtian et al. 2023, Bao et al. 2023).

- **Qi Wang**
- (q_wang2006@126.com)

### Anthony [“Tony”] J. S. Whalley

**Fig. 17:**
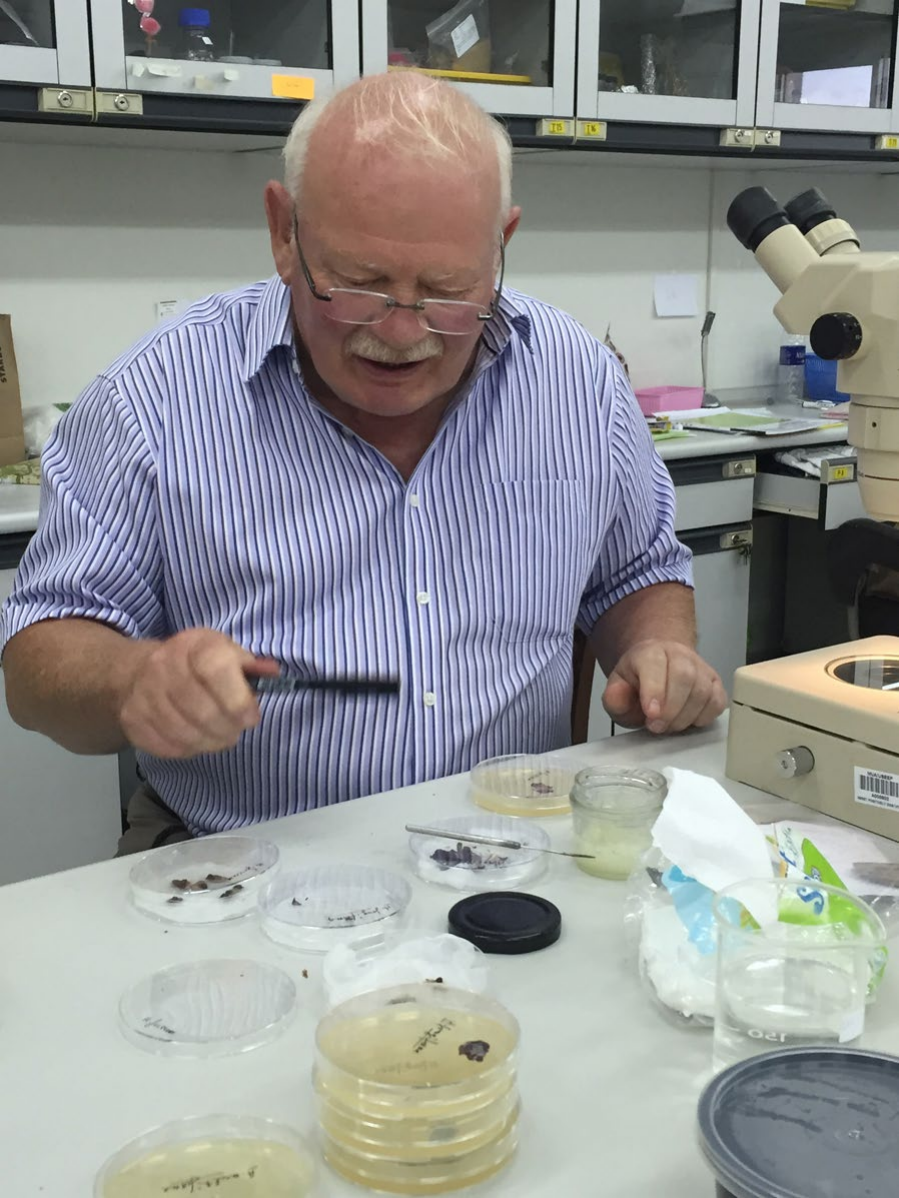
Anthony [“Tony”] Whalley

Tony (Fig. 17) turned 80 on 10 September 2023. I first encountered his name in 1970 when I was confronted with a copy of his University of Liverpool PhD thesis which was being examined externally by Colin Booth (1925–2003); he wondered what I thought of his numerical taxonomic methods! It concerned the taxonomy of *Hypoxlon* in the UK, and that led to a life-time’s dedication to *Xylaricaeae*. His first position was a lecturer at Sunderland Polytechnic in 1971, and in 1977 he moved to Liverpool Polytechnic which then morphed into Liverpool John Moores University where he was made Professor of Mycology in 1991—a position he held until his retirement in 2009 when he was appointed Emeritus.

While taxonomy was always his primary interest, he soon started to also explore the family’s rich chemical diversity and the bioactivity of some of the novel compounds discovered, which involved a close working and extraordinarily productive relationship with chemist Raymond “Ray” L. Edwards (1931–2015). He was also inspired by Jack D. Rogers (1937–2021) of Washington State University with whom he also developed a close working relationship. Tony became especially interested in tropical members of the family, particularly in South-East Asia, forming links with mycologists in Thailand in particular. In 1998 he was awarded the degree of DSc by the University of Liverpool for his researches. He recently prepared a reflection on explorations into *Xylariaceae*, along with his Thai colleagues (Suwannasai et al. 2023).

Tony’s collaboration with mycologists in Thailand continues to prove remakably productive, and has already involved him in supervising over 20 PhDs, many of which involved the students spending some time in Liverpool. He was also the face of the Bitish Mycological Society for several decades, serving as General Secretary, Chairman of Publications, Treasurer, and latterly its Advisor on International Relations. In all these activities, he has been strongly supported by his Welsh-speaking wife Margaret who has been so active in promoting mycological education in the UK and internationally.

We wish Tony and his family all the best for the future, and long may they continue to enjoy their home and garden in, appropriately, the village of Mold in Cheshire.

## BOOK NEWS

### S’initier et se perfectionner à l’étude des Discomycètes. ***By René Dougoud. 2023. Marigny, France: Ascomycete.org. Pp. 82. ISBN 978-2-9875555-0-8. Price: € 20.***

(Fig. 18).

**Fig. 18:**
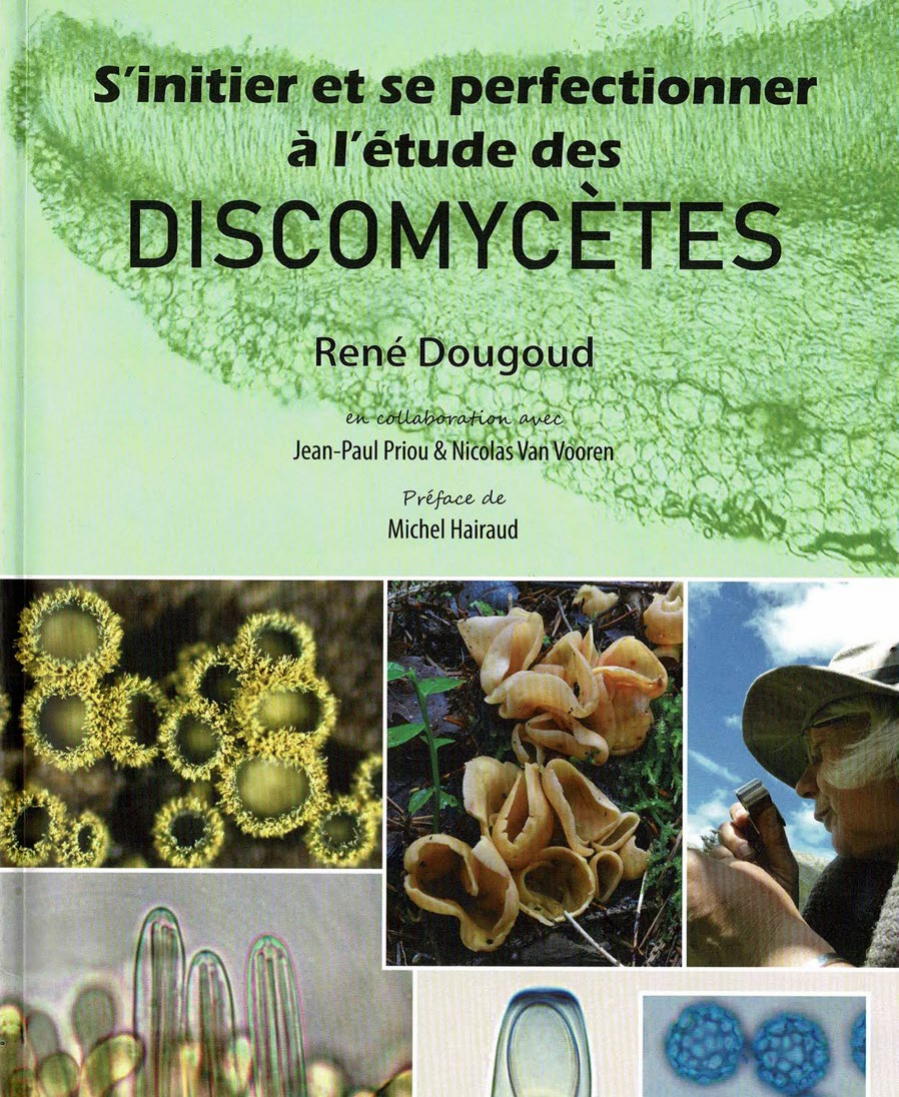
L’Étude des Discomycetès (2023)

This superbly illustrated full-colour manual really brings discomycete fungi to life. An overview of the recognized classes is followed by an introduction to their ecology and roles as parasites, symbionts, and endophytes. The major part of the work, however, is focussed on microscopic features and the use of particular staining techniques and the histological features they reveal. The recipes for a remarkable number of stains and the details they reveal, extracellularly and intracellularly, are provided by a combination of coloured diagrams and photographs. Special attention is given to the variety of paraphyses, ascus tips, ascospores, ascospore sheaths, excipular crystals, excipular hairs, and tissue types (with a valuable key to six textura categories). Information on the use of damp chambers and the preservation and documentation of fungarium specimens is also provided.

This is a real testimony to what a wealth of characters can be revealed in fresh material of discomycetes through the so careful pioneering staining and elegant microscopy launched by Hans-Otto “Zotto” Baral as ‘vital taxonomy’ now some 30 years ago.

Although in French, this little book merits an international readership and I do encourage those who work also on other groups of field-collected ascomycetes to try some of the methods so neatly presented here—and at such an affordable price.

### **Blight: fungi and the coming pandemic.***By Emily Monsoon. 2023. New York: W. W. Norton. Pp. xvii* + *353. ISBN 978–1-324–00701-2. Price: US $ 28.95. £ 14.99 (hbk).*

(Fig. 19)

**Fig. 19:**
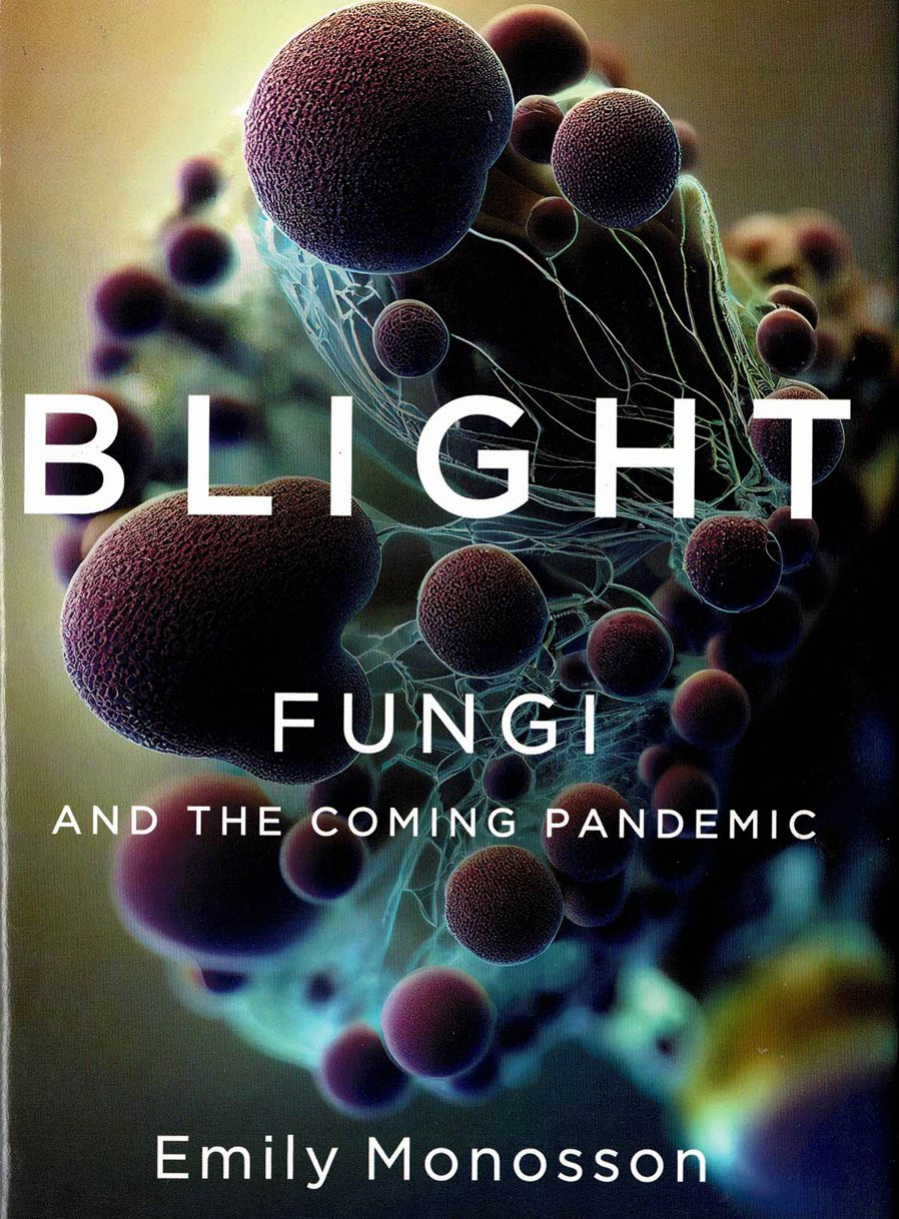
Blight: Fungi and the coming pandemic (2023)

The fly-leaf introduces this book as “a prescient warning about the mysterious and deadly world of fungi—and how to avert further loss across species, including our own”. It was evidently largely stimulated by the “Emerging fungal threats to animal, plant and ecosystem health” warning of Fisher et al. (2012) that spurred the author to embark on a mission to bring these potentially globally important issues to a wider public. This involved her interviewing numerous mycologists and an extraordinary scouring of pertinent literature to put meat on the bone of the messages she was so keen to disseminate—there are five pages of Acknowledgements and 195 notes to original publications or expanding on particular points.

It is not all doom-and-gloom, however, as such a book could easily be. It is divided into two parts, Consequences and Resolution, each with five chapters. Consequences starts with the story of the emergence and spread of *Candida auris* and includes many fascinating insights that were quite new to me. The case of *Batrachochytrium dendrobatidis* follows, as an example of Extinction, including the experiences of Karen Lips who first noted the decline of frogs in Costa Rica, and the subsequent discovery of its origins far away on the Korean peninsula. Plant pathogens illustrate Catastrophe, citing various rusts, including *Cronartium ribicola* and the role of its White Pine and *Ribes* alternate hosts, and *Cryphonectria parastica* on *Castanea*. Effects on Sustenance are demonstrated by Fusarium wilt of bananas and current expansion of *Fusarium odoratissimum* which can infect the Cavendish cultivar resistant to *F. oxysporum* f.sp. *cubense* on which growers have come to depend; a case which demonstrates the risk of relying on monocultures of single genotypes. The concluding chapter in this section deals with *Pseudogymnoascus destructans* on bats in North America, which evidently came from Europe, with considerable detail on its spread in hibernacula.

As examples of Resolution, the first example chosen is of Resistance breeding against the effect of *Cronartium ribicola* on White Pines. Maintaining Diversity is recognized as important as in the arms-race case on *Puccnia graminis* on wheat, where the rust is continually changing and there have been alarms over the Ug99 race. I was also pleased to see the relatively little-known case of azole-resistant *Aspergillus fumigatus* featured which evidently was selected for as a consequence of the use of fungicides by Dutch bulb producers. Resurrection as a way of recovery by plant breeding programmes and genetic engineering is seen as a way forward, but she rightly stresses that it is retaining as much genetic diversity as possible in a crop which is of the utmost importance. While one cannot argue with the principle and need for Certification to try and keep particular diseases out of a country, the next route to resolution proposed, the extent to which that can ever be effective against unknown disease threats is uncertain. The controls being put in or already in place in the USA are described, and I was pleased to see the potential role of wildlife movements, particularly birds, in spreading fungi recognized. Personally, I would have given more emphasis to the consequences of humans, their goods, and their vehicles of all kinds moving around the globe by land, sea, and air—especially with the current popularity of ecotourism in remote environments.

The final chapter is appropriately on Responsibility, but in that the almost impossible task of ensuring even spacecraft are mould-free and that returning astronauts and space vehicles do not bring alien microbes back to Earth. She looks towards a “global census of *all* known fungal species in the environment” and “developing databases of fungal genomes” (p. 195); while mycologists may enthusiastically concur, such aspirations will never be realized unless there are major changes in research priorities in the developed world.

As will be evident from the above, the examples selected are predominantly North American. Discussions of cases like *Hymenoscyphus fraxinea* on ash, emerging diseases on rice, *Phytophthora ramorum* on conifers in Europe, *P. cinnamomi* especially in Australia, *Ophiostoma novo-ulmi* on elms, and the recent appearance of *Cryphonectria parasitica* on sweet chestnut in the UK, might have broadened its appeal. Nevertheless, hopefully, this fact-packed and carefully researched book will be widely read and be something of a wake-up call not only to a public which too often seems to be unaware of the threats fungi can pose to our well-being, but also those concerned with our environment, food supply, and health.

### Ganoderma diseases of tropical crops. *By Carmel A. Pilotti and Paul D. Bridge. 2023. Wallingford: CAB International. Pp. viii + 177. ISBN 978-1-80062-076-6 (hbk), 978-1-80062-078-0 (ebk). Price: £ 105 (hbk and ebk)*

(Fig. 20)

**Fig. 20:**
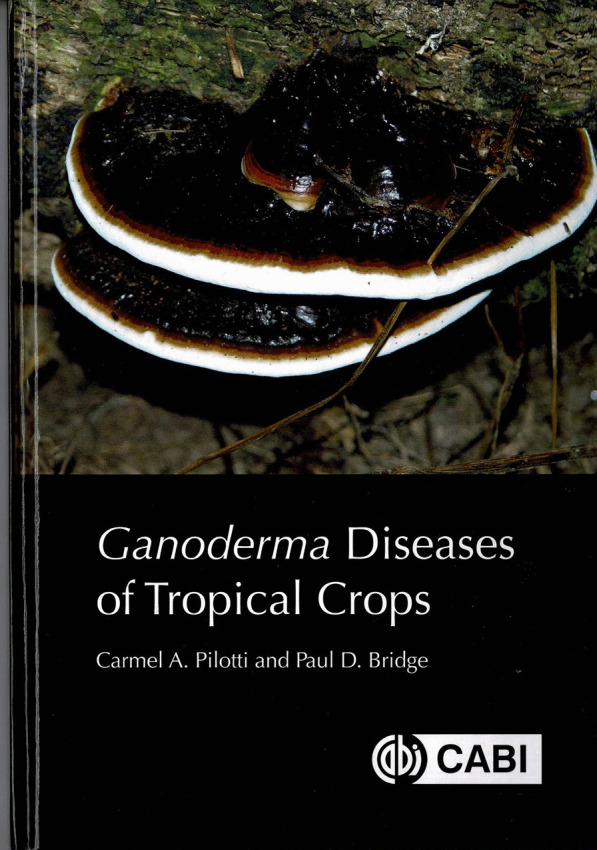
Ganoderma Diseases of Tropical Crops (2023)

European mycologists generally associate *Ganoderma* species with aged trees such as beech and oak, and some conifers, rather than root and stem rots in tropical perennial crops such as oil palms. Others may be aware of it because of species which are widely recognized as having human health benefits. It may come as something of a shock to find that there are around 400 species described, although many of those are synonyms of others, and that some can be so important in tropical situations. The background to this work was research in the 1990s into just what species were causing major losses in oil palm plantations in South-East Asia, more particularly in Indonesia and Malaysia. This has now been broadened out to provide a comprehensive treatment of the taxonomy of the species involved and the diseases they cause in particular crops.

The species of the genus as a whole fall into some ten subgeneric ITS clades, and I am relieved that no-one has yet made the mistake of recognizing these as separate genera which would hardly advance scientific communication. The morphological and microscopic features are described, and I especially liked the scanning electron micrographs (SEMs) of selected basidiospores that really show the marked differences in surface ornamentation. Particular emphasis is paid to molecular approaches and previous studies are reviewed, but the authors caution that ITS sequences “can prove unreliable in some instances” (p. 18) as it has become evident that there can be within-species variation. The key species of concern here fall into three main groups: the *G. lucidum* complex, *G. lingzhi* and related species, and *G. applanatum*/*G.australe* lineages. Some specific primer/PCR methods including multiplex PCR and RFLP comparisons are, however, available. The species of especial concern on palm hosts prove to be *G. boninense* (Asia and Oceania), *G. ryvardenii* (Africa) and *G.zonatum* (Colombia and the USA).

The biology, life-cycle, and genetics are described, and I was surprised to see evidence of such strong antagonistic reactions between dikaryons of different *G. boninense* individuals; these can apparently be used to infer patterns of disease spread. As regards disease detection, methods used range from air sampling to remote sensing and laboratory diagnosis by not only molecular but also immunological ones. There are separate chapters on the diseases and their control in oil palm, coconut palm, other palms and other monocots, *Acacia*, beverage crops (tea, cocoa and coffee), rubber, and other tropical crops; I found the full list of commercially important hosts quite surprising (p. 5).

The taxonomy of the species of major concern on palms are first considered separately, with nomenclatural information and details of the type materials, detailed descriptions, including variations in spore sizes. A similarly detailed treatment of those pathogenic on woody crops follows, and then one on “other species reported pathogenic to tropical commodity trees” with data on 36 species—including a most useful table comparing their spore shapes and sizes.

The book concludes with a chapter on common issues for improved management, covering taxonomic concepts and reference materials as well as issues of infection, ecology, interactions, mediation, and management. The list of References is an amazing 34 pages in small type.

It was a real pleasure to see a well-presented work blending pathology and taxonomy so neatly together and this will surely be regarded as both a landmark in the study of *Ganoderma* diseases in the tropics and a significant contribution to the clarification of their taxonomy.

### Fungi and food spoilage. *By John I. Pitt and Ailsa D. Hocking. 2022. Cham, Switzerland: Springer Nature. 4th Edn. Pp. xxi* + *645. ISBN 978-3-030-85638-0 (hbk), 978-3-030-85640-3 (ebk). Price: £ 129.99 (hbk).*

(Fig. 21)

**Fig. 21:**
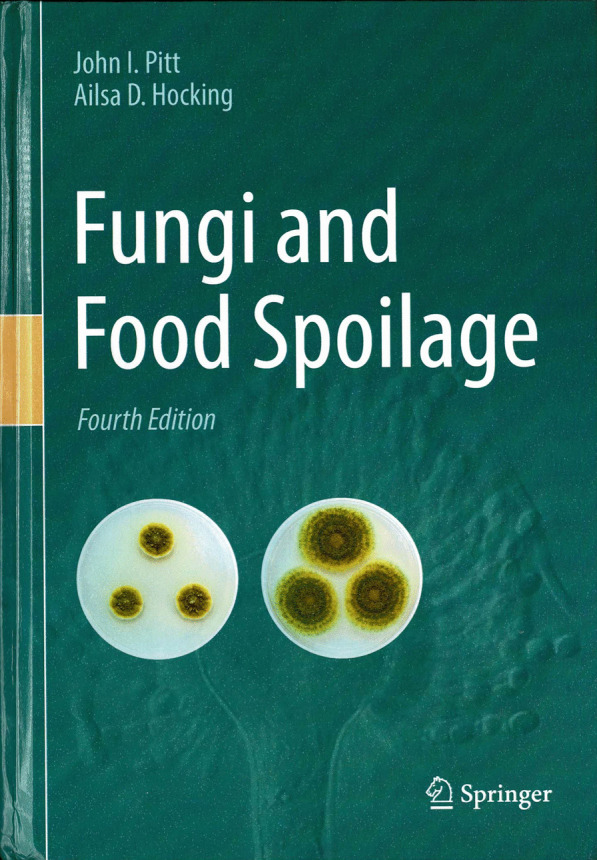
Fungi and Food Spoilage (2022)

Since the first edition of this book was published by Academic Press (London) in 1985, it has become the *vade mecum*, the go-to reference work, on issues of food spoilage involving fungi. Each of the next two editions, in 1997 and 2009, were extended and improved, and after an interval of 13 years a new edition was clearly due. The fourth edition has swelled by a rather modest 126 pages, but what really makes it stand out is that many of the photographs of Petri-dish cultures are now in colour—a most useful addition.

The structure largely follows the arrangement of the third, the principle sections being on the: Ecology of food spoilage; Naming and classifying fungi; Methods for enumeration, isolation and identification; Keys and generic accounts (five chapters which form the major part of the book); Fresh and perishable foods; Spoilage of stored, processed and preserved foods; with a new chapter on Mycotoxins.

The keys and generic and species accounts are the meat of the book and really its special feature, making up 446 pages, almost 70% of the whole. For each species there is information on the main synonyms, detailed descriptions including information on growth rates on different media at different temperatures (an approach the authors pioneered as a practical aid to species identification in *Penicillium* in particular), a discussion of the taxonomy and differentiation from similar moulds, physiological features, mycotoxin production, ecology (including issues caused), key references, and especially photographs of cultures (many in colour) and key microscopic features (mostly in differential interest contrast). This makes the book of enormous benefit for information on numerous fungi not just to do with food, but isolations or occurrences on any substrate. Indeed, in my own work the information on growth rates of some moulds at different temperatures has been of assistance in determining post-mortem intervals of human cadavers.

One point which I do feel is to be regretted is that some of the segregate genera of *Aspergillus*, notably *Emericella* (e.g. *A. nidulans*) and *Neosartorya* (e.g. *A. fumigatus*) are employed, contrary to the recommendations of the International Commission of *Penicillium* and *Aspergillus,* and so out of step with the practice now being followed by those working in other fields of applied mycology. This view has also led to them making four new combinations (three into *Emericella* and one into *Neosartorya*) and also some new and corrected typifications (p. 611).

The new Chapter on mycotoxins is especially welcome, bringing together practical issues such as exposure and management of risk as well as the adverse effects they can cause.

This is a must-have for all who deal with spoilage issues involving moulds, and not only on foodstuffs, and it is pleasing to have this fresh edition with information added from work over the 13 years that has passed since the third edition. Sadly, John did not live to see the new edition come out as he died on 23 March 2022 aged 85 (see *IMA Fungus* **14** (1): 13–14, 2023) and Alisa, his colleague for over 40 years, saw it though its final stages of production. It also marks a passing of an era in two other ways: the North Ryde Laboratory where they worked has closed and the fungal culture collections (FRR) built and used in this work have been transferred to the New South Wales Department or Primary Industries located in Orange; and the much appreciated courses he gave on food spoilage fungi (sometimes with colleagues) around the world over so many years have also come to an end.

The book will, however, remain as a treasured testimony to the work of a most remarkable man who devoted most of his life to improving our understanding of food spoilage issues involving fungi, and particularly the characterization of the species involved.

### Evolution of fungi and fungal-like organisms. *Edited by Stephanie Pöggeler and Timothy James. 2023. Second edition. [The Mycota. vol.14.] Cham: Springer Nature. Pp. xiv* + *322. ISBN 978-3-031-29198-2 (hbk), 978-3-031-29199-9 (ebk). Price: £ 159.99 (hbk).*

(Fig. 22).

**Fig. 22:**
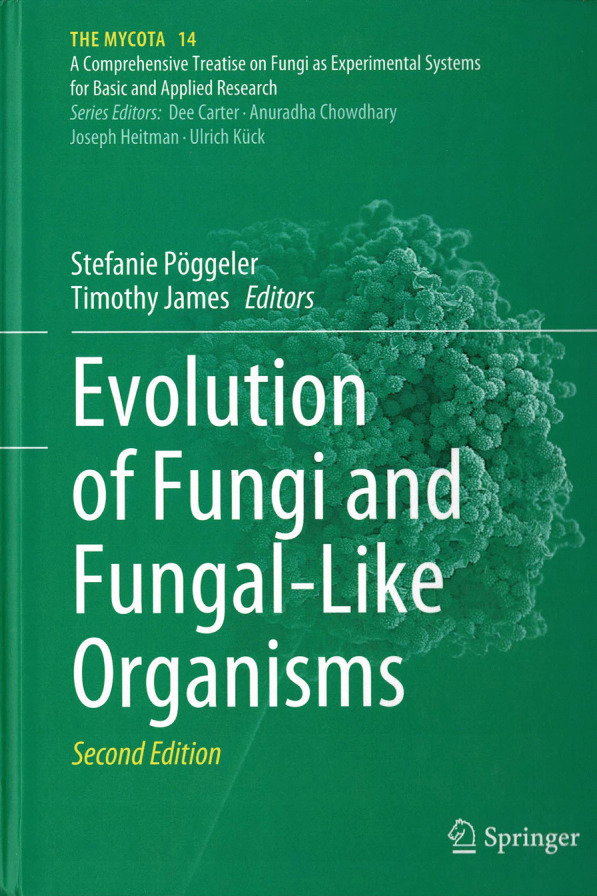
Evolution of Fungi and Fungal-like Organisms (2023)

Although described as a second edition, this is essentially a new book in the same broad subject area; only two of the 13 article titles appeared in the 2011 edition, and they have been thoroughly revised and have different authorships. In view the advances made in our understanding of fungal evolution at the molecular level since that time, especially the increasing number of genome sequences, that is hardly surprising.

I found the article starting the volume of particular interest. An overview of the *Opisthokonta* “supergroup” which comprises the *Fungi* and *Metazoa* (animals), and draws attention to he question as to whether *Opisthosporidia* (i.e. *Aphelidea*, *Cryptomycota, Microsporidia,* and nucleated *Amoebae*) should be included in the *Fungi* or not; together those groups are referred to as *Holomycota,* although using the phylum indicating suffix”-*mycota*” is hardly appropriate for a superkingdom! The *Holomycota* are a sister group to the *Holozoa*, which includes a huge range of unicellular and many flagellated organisms generally unfamiliar to mycologists. Just where to draw the lines is likely to be debated for many years yet and can be expected to be refined as more genomic data become available. It was also pleasing to see a contribution on mitochondrial genomes (“mitogenomes”) as phylogenetic inferences based on them have become rather eclipsed by work on nuclear sequences. They do, however, have conserved ancestral elements and act as something of a test for the robustness of relationships, especially at the ordinal level. There is increasing support for the original endosymbiotic bacterium that gave rise to the mitochondria in fungi being infected with a particular phage.

Two chapters focus on genome evolution in plant pathogens. One explores factors such as drivers of diversification, the role of transposable elements and epigenetic modifications and the other on range expansions and host-switching in mildews, rusts, and smuts. I was also fascinated by the contribution on activation of secondary metabolites as it becomes evident that many fungi have sets of biosynthetic gene clusters that remain silent until there is a stimulus that switches them on; this is of immense importance as it is pertinent to mycotoxin production as well as species interactions in nature.

Of general interest is the contribution aiming to provide a new estimate of global fungal diversity by high-throughput sequencing of environmental samples and using the ITS region and 97% sequence similarity as the species-level threshold. Using data from the GlobalFungi database containing metabarcoding data from a staggering 36,684 samples of fungal communities containing 1.1 billion sequence records, Petr Baldrian and colleagues provide an estimate of 6.3 million species of fungi—more than double the 2.5 million figure arrived at in the latest *State of the Worlds Plants and Fungi* assessment (see earlier in this edition of MycoNews) published after *The Mycota* volume appeared.

Macrofungi hardly feature in this edition except for a fascinating chapter on paedomorphosis in sequestrate basidiomycetes. It is argued that the key processes involved are either the retention of juvenile features (neoteny) or the onset of sexual maturity while in a morphologically immature stage (progenesis). While there are many examples, the genomic basis for these remains to be explored and promises to be an intriguing topic for the future.

I have concentrated here on chapters which I found of particular interest myself, but draw attention also to others which address *Dictyostelium* morphogenesis, *Mucor* dimorphism and pathogenicity, mycoviruses, bacterial endosymbionts of *Mucoromycota*, fungi and their environmental micropredators, and bioluminescent fungi.

The Editors are to be congratulated on putting such a great selection of topics together!

### Plant Relationships: fungal-plant interactions. *Edited by Barry Scott and Carl Mesarich. 2023. Third edition. [The Mycota. vol. 5.] Cham: Springer Nature. pp. xxii* + *462. ISBN 978-3-031-16502-3 (hbk), 978-3-031-16503-0 (ebk). Price: £ 199.99 (hbk).*

(Fig. 23)

**Fig. 23:**
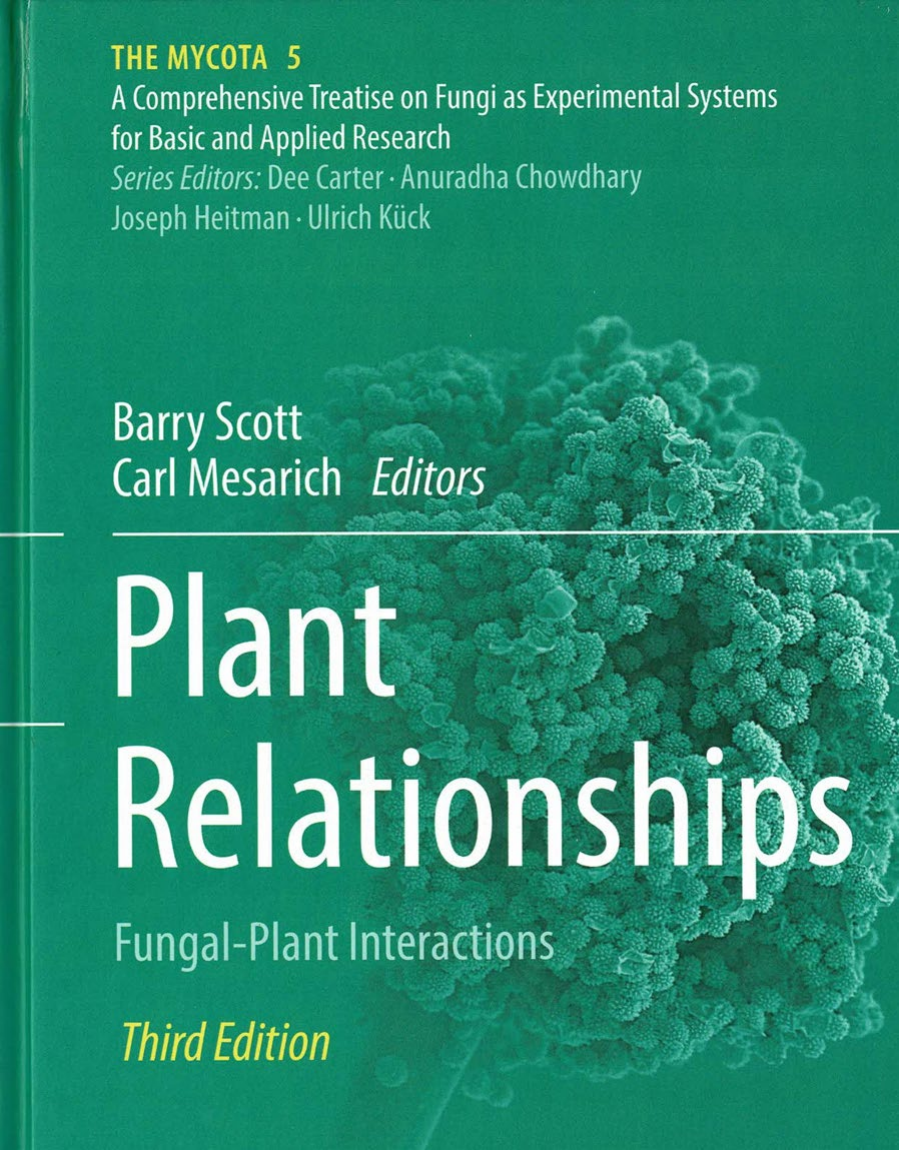
Plant Relationships (2023)

This edition has a mixture of chapters, some of which are broad in scope and so will be of interest to a wide range of mycologists, while others focus on particular cases. With regard to plant pathogens, there is a fascinating account of the role of extracellular RNAs in overcoming the species boundary between a fungus and plant host; these RNAs can be associated with extracellular vesicles and modify host responses to infection. There is also an update on the role of MAP kinase (MAPK) pathways in modifying or regulating infection and developmental processes. Ambient pH sensing and adaptation is reviewed and the ability of fungi to modify and maintain the pH of the surrounding host tissues. Volatile organic compounds are now attracting more attention, and one contribution here explores their role in *Trichoderma* species interactions with plant hosts; they can evidently promote plant growth and immunity. Also coming to the fore today are questions over epigenetic controls of gene expression, so I was pleased to see an in-depth review of these in relation to chromatin organization remodelling and impacts on the regulation of genes involved in host colonization.

Three kinds of mutualistic interactions are considered. First, advances in our understanding of the genomes of arbuscular mycorrhizal fungi (AMF) and their implications for our understanding of AMF biodiversity, adaptation, and evolution; epigenetic regulation again comes to the fore. Second, seed endophytes, in effect heritable symbionts transmitted directly from a host plant to its progeny. The importance of endophytic fungi seems to have been underestimated, and I was interested to see the case of an *Epichloë* gene transferred to a grass host to which it conveys disease resistance. The third mutualism considered is that of lichen-forming fungi. Rosmarie Honegger has updated her superb contribution from the previous edition; it has almost doubled in length from 26 top 50 pages, profusely illustrated with stunning colour photographs and also scanning electron micrographs. Aspects developed here include sections on the lichen bacterial microbiome, endolichenic fungi, viruses, lichenicolous fungi, interactions with animals (as food and in propagule dispersal) and mimicry. Secondary metabolites produced by lichen fungi are now moved to an independent chapter; advances in our understanding of the biosynthetic pathways involved has improved immensely in recent years as a consequence of genome sequencing; some species may have as many as 120 biosynthetic gene clusters, and genetic engineering has enable some of these to be expressed in cultures of yeasts and other filamentous fungi.

Other contributions in this third edition, however, concern advances in our understanding of aspects of particular diseases, especially ones facilitated by genomic and genetic studies. There are ones devoted to aspects of *Botrytis cinerea, Fusarium fujikuroi, F. oxysporum, Magnaporthe oryzae, Parastagonospora nodorum, Ustilago maydis*, and *Zymoseptoria*. There are also prescient concluding chapters, one concerning the risks posed by *Puccinia* species epidemics to major world crops (barley, maize, oats, sorghum, sugarcane, and wheat), and another the threat *Magnaporthe oryzae* and its pathotypes pose to global food security as so much of the world depends on rice.

### Finding the mother tree: uncovering the wisdom and intelligence of the forest. *By Suzanne Simard. 2022. Penguin Books, London. pp. 348, ISBN 978-0-141-99028-6. Price: £ 10.99 (pbk).*

(Fig. 24)

**Fig. 24:**
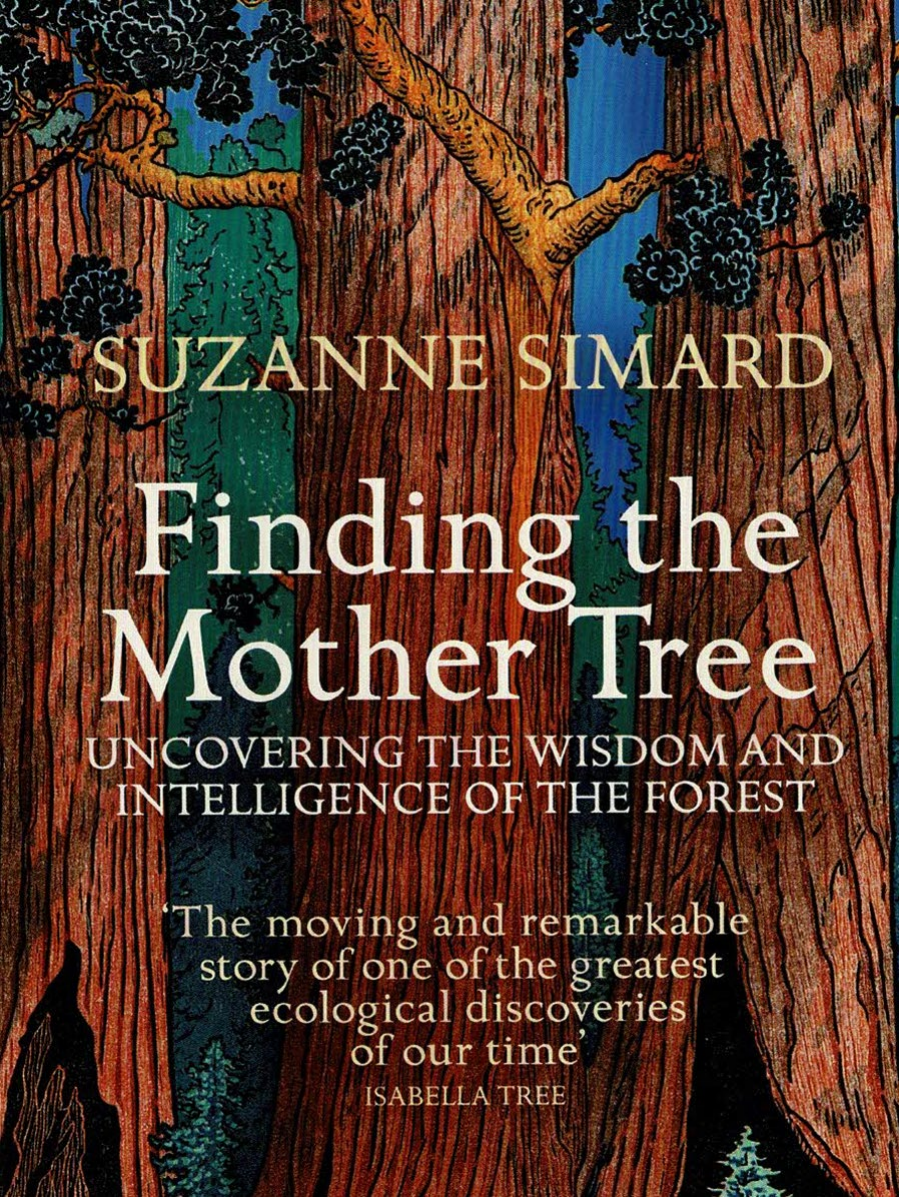
Finding the Mother Tree (2022)

This is a remarkable personal story, and also a scientific memoir, and an insight into the author’s pioneering work to investigate the mycorrhizal relationships and links between trees in the forests of British Columbia. She explains how she started working with foresters and making observations on regrowth after clear-felling, and came to realize that the emerging or newly planted trees grew much better when there were uncut trees nearby; “mother trees” that were able to pass nutrients through mycorrhizal networks to the developing ones.

Her proposals on leaving some trees during fellings went unheeded by the timber companies, although she could see the beneficial effects in the field through repeated visits to different sites. She also came to recognize different mycorrhizal fungi from the growth on roots she unearthed and that they were the same on those of the “mother tree” as well as the newly planted trees.

The work was followed up with elegant experimental studies, leading to a substantial series of reports and then scientific publications from around 1990 onwards. These included using carbon-13 from cylinders to establish movements of sugars from the elder to newly planted trees. The so-familiar phrase, “the wood-wide web” actually came from a comment in *Nature* which featured some of her work in 1997. Suzanne obtained a position at the University of British Columbia in 2005 that greatly facilitated such studies, and where she is now Professor of Forest Ecology. Unusually for a book aimed at a general audience, detailed lists of references to the pertinent scientific literature are collected at the end of the book so that these can be followed up. It is frustrating in some popular science works not to know where the work discussed appeared.

There are a couple of signatures of some lovely and highly pertinent colour photographs that really give an impression of the splendour of the West Coast forests, and also many half-tones scattered through the text, some catching personal and family moments.

Suzanne also provides much personal information and reflections from her earliest days growing up in the Monashee Mountains, learning about nature from her grandmother, and hiking and picnicking in the forests. She also reflects on the practices and views of the indigenous peoples which she learnt to greatly respect. It is also clear that while she went through some difficult personal times, raising her daughters and later battling with cancer, she was always determined to make sure her messages were widely known and acted on, and that is something this book will help ensure.

An inspiring story and one which demonstrates how much an individual can do to challenge an established view, undertake work to redirect actual practices, inform and educate, so changing entrenched opinions—and in this case to headline the importance of keeping mature trees in forestry projects. The “wood-wide web” has now come into almost general use greatly in response to her determination and pioneering work.

## NOTICES

1. *MycoNews* is compiled by David L. Hawksworth as Editor-in-Chief, and to whom all material for consideration for inclusion in *MycoNews* should be sent directly by e-mail.
2. Books for possible coverage in the Book News section should be mailed to David L. Hawksworth at Milford House, 10 The Mead, Ashtead, Surrey KT21 2LZ, UK; works issued only as e-books are not normally included. Reviews prepared by others will also be considered if sent to him.
3. Reports of new genome sequences intended for inclusion in the *Fungal Genomes* compilation should be sent directly to Senior Editor Brenda Wingfield as e-mail attachments and not submitted through Editorial Manager.
4. All unsigned items in *MycoNews* can be attributed to the compiler, David L. Hawksworth.
5. The compiler is particularly indebted to Pedro W. Crous for permission to adapt some material from the *IMC12 Newsletters* for inclusion in this edition of *MycoNews*.
