# Correction: MycoNews 2023: Editorial, news, reports, awards, personalia, and book news

**DOI:** 10.1186/s43008-024-00143-y

**Published:** 2024-05-29

**Authors:** David L. Hawksworth

**Affiliations:** 1 Trait Diversity and Function, Royal Botanic Gardens, Kew, Surrey TW9 3DS UK; 2 Department of Life Sciences, The Natural History Museum, Cromwell Road, London, SW7 5BD UK; 3 Jilin Agricultural University, Changchun, 130118 Jilin Province China; 4 Geography and Environmental Science, University of Southampton, Highfield Campus, Southampton, SO17 1BJ UK

**Correction** **:** **IMA Fungus 15, 1 (2024)**

**https://doi.org/10.1186/s43008-024-00139-8**

After publication of this article (Hawksworth 2024), it was reported that in the legend of Fig. 13, ‘Takashi Matsushima (1930–1923)’ should have read 'Takashi Matsushima (1930–2023)’.

The original article has been updated.
